# Rapid Fabrication of High-Performance Flexible Pressure Sensors Using Laser Pyrolysis Direct Writing

**DOI:** 10.1021/acsami.3c04290

**Published:** 2023-07-31

**Authors:** Shaogang Wang, Qihang Zong, Huiru Yang, Chunjian Tan, Qianming Huang, Xu Liu, Guoqi Zhang, Paddy French, Huaiyu Ye

**Affiliations:** † Faculty of EEMCS, Delft University of Technology, Mekelweg 4, 2628 CD Delft, The Netherlands; ‡ School of Microelectronics, Southern University of Science and Technology, 518055 Shenzhen, China

**Keywords:** flexible pressure sensor, UV laser, laser direct writing, continuous laser pyrolysis, PDMS, micro-truncated pyramid

## Abstract

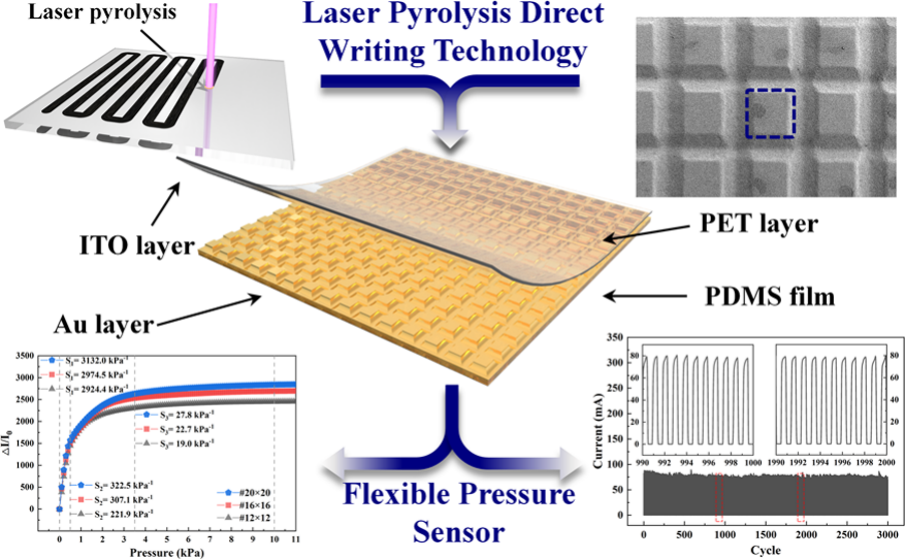

The fabrication of flexible pressure sensors with low cost, high scalability, and easy fabrication is an essential driving force in developing flexible electronics, especially for high-performance sensors that require precise surface microstructures. However, optimizing complex fabrication processes and expensive microfabrication methods remains a significant challenge. In this study, we introduce a laser pyrolysis direct writing technology that enables rapid and efficient fabrication of high-performance flexible pressure sensors with a micro-truncated pyramid array. The pressure sensor demonstrates exceptional sensitivities, with the values of 3132.0, 322.5, and 27.8 kPa^–1^ in the pressure ranges of 0–0.5, 0.5–3.5, and 3.5–10 kPa, respectively. Furthermore, the sensor exhibits rapid response times (loading: 22 ms, unloading: 18 ms) and exceptional reliability, enduring over 3000 pressure loading and unloading cycles. Moreover, the pressure sensor can be easily integrated into a sensor array for spatial pressure distribution detection. The laser pyrolysis direct writing technology introduced in this study presents a highly efficient and promising approach to designing and fabricating high-performance flexible pressure sensors utilizing micro-structured polymer substrates.

## 1 Introduction

With the rapid development of the Internet of Things (IoT), more and more sensors are being integrated into daily life and industrial production as an important sensing medium. Flexible pressure sensors have gained considerable attention due to their potential applications in health monitoring,^1^ human–machine interaction,^2^ electronic skin,^3^ soft robotic,^4^ etc. To satisfy the demands of these applications, flexible pressure sensors should not only possess high sensitivity, wide detection range, fast response time, excellent repeatability, and robust reliability but also offer the advantages of low cost, high scalability, and easy fabrication. Flexible pressure sensors are generally categorized into four types based on their sensing mechanism and signal transmission mode: piezoresistive,^5−7^ piezoelectric,^8−10^ capacitive,^11,12^ and triboelectric.^13,14^ In the case of piezoresistive flexible sensors, applying external forces to their sensing material and device structure induces local strain in the force-bearing area. Consequently, the resistance network, consisting of material resistance and contact resistance within the sensor, changes with the localized strain, enabling the detection and identification of pressure.^15,16^

Advanced materials, including carbon nanotubes,^17^ carbon nanofibers,^18^ graphene,^19,20^ MXene,^21,22^ metal nanowires,^23^ and nanoparticles (NPs),^24^ have been widely applied as sensing elements to create electrical percolation pathways. In addition, flexible materials such as poly(dimethylsiloxane) (PDMS),^25^ polyimide (PI),^26^ poly(ethylene terephthalate) (PET),^27^ polyethylene (PE),^28^ and polyurethane (PU)^29^ serve not only as flexible substrates for active materials but also as flexible electrodes, which can be fabricated through methods like infilling,^30^ coating,^31^ and modification.^32^ Therefore, introducing advanced sensing materials or constructing effective microstructures has been considered in numerous reports as two comprehensive strategies to obtain high-performance flexible sensors.^33,34^ Especially using PDMS as a substrate material for creating surface microstructures is widely regarded as a promising strategy for fabricating flexible pressure sensors due to its exceptional flexibility, transparency, and biocompatibility.^2,35,36^

For the fabrication of flexible sensors with various microstructures, the template method is a widely adopted manufacturing technology. This method involves casting or coating uncured elastic materials onto molds, such as silicon wafers,^37^ abrasive paper,^38^ natural leaves,^39^ silk,^40^ etc., to transfer patterned microstructures. In particular, traditional photolithography has been widely employed as a precise and efficient method for fabricating well-designed silicon molds used in the production of high-performance flexible pressure sensors.^41^ However, this method requires a strictly controlled fabrication environment, precise equipment, and complex processes to maintain fabrication accuracy. Hence, it is still a significant challenge to design and fabricate flexible pressure sensors that combine high sensitivity and wide detection range with low cost and simple process. To date, the potential applications of laser direct writing (LDW) technology in surface modification,^42,43^ nanomaterial synthesis,^44,45^ and microfluidic device design^41,46^ have attracted considerable interest. Laser direct writing enables the fabrication of diverse functional devices without requiring photomasks. Particularly in the field of microstructure fabrication, it allows for the creation of not only two-dimensional surface microstructures^47,48^ but also three-dimensional (3D) microstructures.^49,50^ Compared to traditional physical and chemical fabrication methods, laser direct writing technology has absolute advantages in terms of efficiency, precision, controllability, and flexibility.

Herein, we develop an efficient and promising approach for designing and fabricating flexible pressure sensors with a micro-truncated pyramid array using laser pyrolysis direct writing (LPDW) technology. To optimize the fabrication parameters, we summarized the realization conditions for continuous laser pyrolysis (CLP), such as the average laser power, pulse repetition frequency, and laser focal distance. The mechanism of continuous laser pyrolysis was then explored and investigated through experimental characterization and finite element simulation. On this basis, micro-truncated pyramid structures were fabricated on the surface of a PDMS film by LPDW technology. Subsequently, a thin layer of gold was magnetron sputtered on the surface of the PDMS film with a micro-truncated pyramid array to impart piezoresistive capability. Finally, the PDMS conductive film (Au/PDMS) was assembled with a film consisting of indium tin oxide and poly(ethylene terephthalate) (ITO/PET) to produce the flexible pressure sensor. Performance testing revealed that the pressure sensor exhibited high sensitivity, wide detection range, fast response time, and long-term mechanical durability. Furthermore, the pressure sensor responded quickly to micro-pressure and small-pressure external signals involved in various applications. As a proof of concept, the potential application of the sensor array in real-time detection of spatial pressure distribution was demonstrated.

## 2 Results and Discussion

### 2.1 Realization Conditions of LPDW Technology

**Figure 1:**
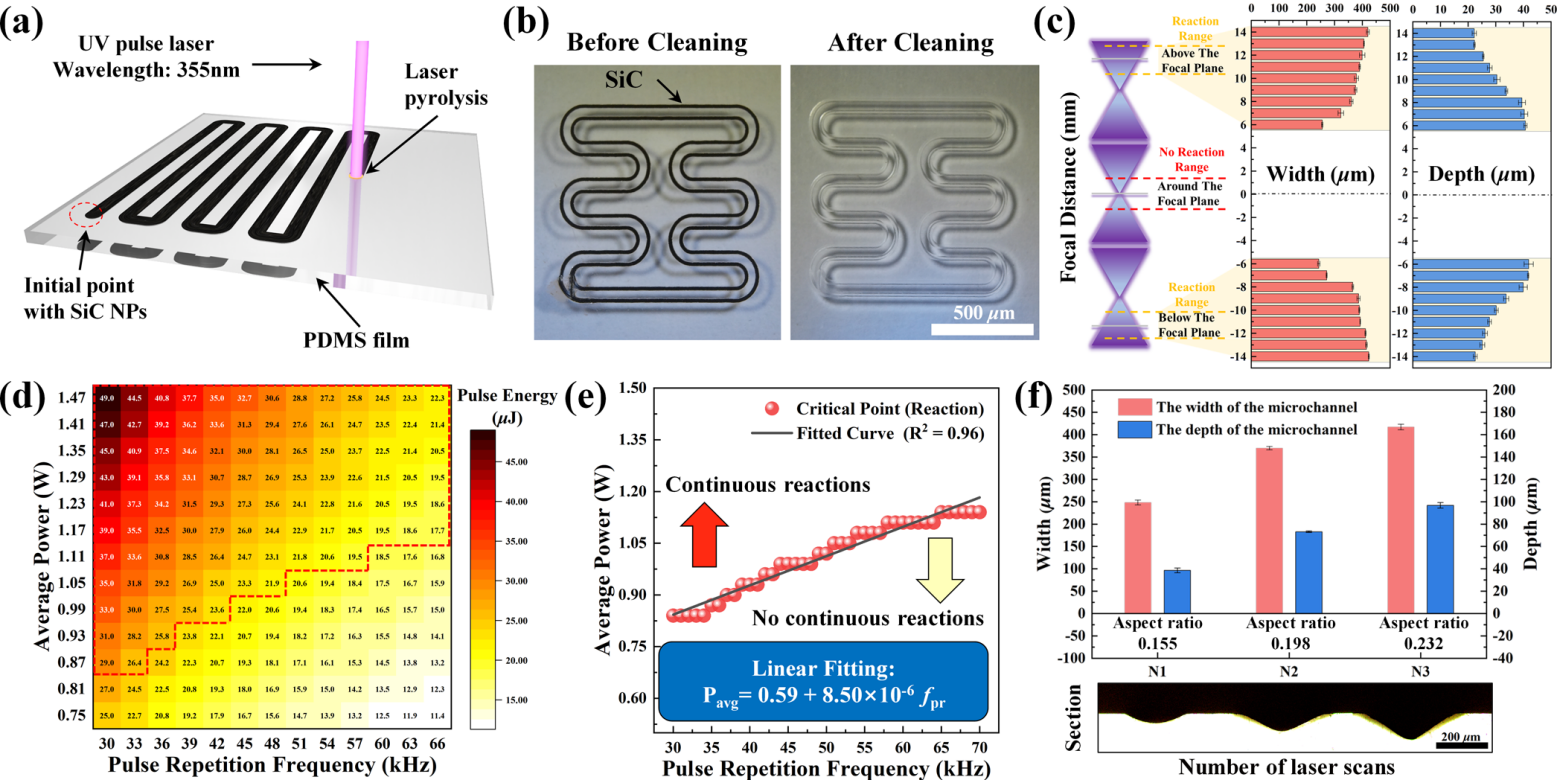
(a) Schematic illustration of the continuous laser pyrolysis (CLP) process. (b) Optical microscope images of the serpentine pattern written by LPDW technology before and after cleaning treatment. (c) Comparison data on microchannel width and depth prepared by LPDW technology at different focal distances (*v*_ss_ = 10 mm/s, *P*_avg_ = 1.5 W, and *f*_pr_ = 40 kHz). (d) Critical realization conditions distribution heatmap for CLP reaction (*d*_def_ = −6 mm). (e) Critical reaction boundary fitting curve of CLP reaction under different *f*_pr_ and *P*_avg_. (f) Comparison of microchannel aspect ratio and cross-sectional morphology under different numbers of laser scans.

It is widely acknowledged that PDMS is a transparent polymer. When a laser is irradiated perpendicularly to its surface, a portion of the laser power is reflected. Moreover, due to the low absorption coefficient of PDMS for ultraviolet (UV) laser light, most of the laser energy passes through the PDMS film without inducing any physical or chemical reactions. However, when opaque 6H-SiC nanoparticles (NPs) are coated at the initial point of the UV pulsed laser scanning path, the photothermal effect of the laser on the 6H-SiC NPs initiates a pyrolysis reaction on the PDMS surface (Figure 1a). This initial laser pyrolysis (ILP) reaction generates 3C-SiC on the PDMS surface, triggering a chain reaction and resulting in a continuous laser pyrolysis (CLP) phenomenon. To maintain a continuous impact of the pulsed laser on the PDMS surface, the pulse repetition frequency must be sufficiently high to ensure the new laser pulse overlaps with the region where the 3C-SiC pyrolysis products are present. In other words, the occurrence of CLP reactions is dependent on the inherent properties of pulsed lasers and takes place iteratively with each pulsed laser irradiation, as illustrated in Figure S1. With a higher pulse repetition frequency at a constant laser scanning speed, the density of overlap between individual circular pyrolysis regions formed by each pulse increases, resulting in a continuous pyrolysis region that approximates a straight line more closely. The comparison video (Video S1) demonstrating the occurrence or absence of the CLP reaction is provided in the Supporting Information.

When combined with advanced laser control software, the laser pyrolysis direct writing (LPDW) technology provides an efficient method for fabricating PDMS surface microstructures. Significantly, we found that the laser pyrolysis products of PDMS exhibited minimal adhesion to the PDMS surface and could be easily removed through ultrasonic cleaning. Figure 1b shows the serpentine pattern written using laser pyrolysis direct writing technology before and after cleaning. Interestingly, we observed that the initiation of CLP reaction on the PDMS surface was restricted to the regions situated significantly above and below the laser focal plane (Figure 1c). In contrast, the regions near the laser focal plane exhibited extremely low sensitivity to the CLP reaction. As a result, within the focal plane range of (−6, 6 mm), the laser failed to induce CLP reactions. This phenomenon can be attributed to the uncertain PDMS surface temperature resulting from the radiation pressure exerted by the laser (see the Supporting Information, Discussions 2.1 for further details). However, upon further defocusing, the CLP reaction was smoothly induced by laser, following the heat flow distribution of the Gaussian heat source. Meanwhile, within the focal plane range of [−6, −14 mm] and [6, 14 mm], the width and depth of the PDMS microchannel increased and decreased, respectively, with increasing defocus. Additionally, at positions distant from the focal plane, the temperature and temperature gradient generated by the laser photothermal effect were insufficient to reach the critical pyrolysis condition of PDMS. Correspondingly, the 3D laser confocal images of the microchannel structure on the PDMS surface at various focal distances are illustrated in Figure S2.

Based on the above phenomenon, we investigated the critical conditions of CLP reaction at different laser average powers (*P*_avg_) and pulse repetition frequencies (*f*_pr_) while maintaining a fixed scanning speed (*v*_ss_ = 10 mm/s). Figure 1d illustrates the reaction conditions under which CLP reaction can occur smoothly, as shown in the area marked with the red dotted line. When the *P*_avg_ was fixed, we observed that a lower *f*_pr_ was more likely to induce CLP reactions. This can be attributed to the pulsed laser mechanism, where reducing the *f*_pr_ results in higher peak power (*P*_peak_) of a single pulse that more easily triggers the CLP reactions. Correspondingly, the schematic illustration of the relationship among *P*_avg_, *P*_peak_, and *f*_pr_ is illustrated in Figure S3. The single pulse energy (*E*_sp_) can be expressed as
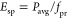
1

Furthermore, when the defocusing distance (*d*_def_ = −6 mm) was fixed, there existed a line relationship between the *P*_avg_ and *f*_pr_ that determined whether the CLP reaction could be induced. Figure 1e shows the energy threshold boundary for a single laser pulse to induce the CLP reaction. The detailed experimental data, as illustrated in Figure S4, are used to analyze the fitted linear relationship, which can be expressed as
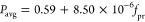
2

After substituting eq 2 into eq 1, the threshold boundary conditions of CLP reactions can be formulated as
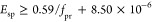
3Surprisingly, after determining the *f*_pr_ and *d*_def_ of the laser, we found that increasing the laser power did not lead to a significant enhancement in the aspect ratio of the microchannel. However, increasing the number of laser scans proved to be an effective approach for improving the aspect ratio of the microchannel (Figure 1f). As the number of laser scans increased from 1 to 2 and 3 (N1, N2, and N3), the aspect ratio of the microchannel increased from 0.155 to 0.198 and 0.233, respectively. Moreover, both the width and depth of the microchannels increased with the number of scans. Cross-sectional optical microscope images of the PDMS revealed that after a single laser scan, the cross section of the PDMS microchannel exhibited a tendency toward a semi-elliptical distribution. However, as the number of scans increased to 2 and 3, the cross section of the PDMS microchannel approached a two-dimensional Gaussian distribution. We attribute this phenomenon to the isotropic extension of the pyrolysis temperature during the CLP reaction (For further details, refer to the Supporting Information, Discussions 2.2). The corresponding scanning electron microscopy (SEM) images of the surface topography after the different number of laser scans are shown in Figure S5. Furthermore, the roughness results for the sidewalls of the microchannel are shown in Figures S6 and S7, which are discussed in detail in the Supporting Information, Discussions 2.3.

### 2.2 Mechanism Analysis of LPDW Technology

**Figure 2:**
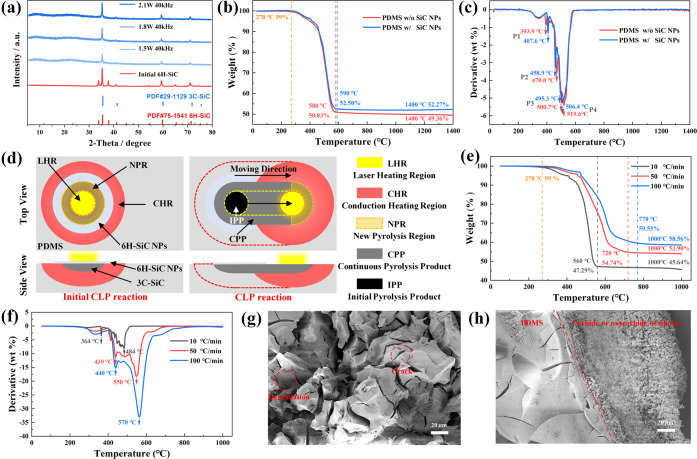
(a) XRD patterns of initial 6H-SiC NPs and 3C-SiC pyrolysis products with different laser average power (1.5, 1.8, and 2.1 W). (b) Thermogravimetric (TG) curves and (c) derivative thermogravimetry (DTG) curves of pristine PDMS and PDMS coated with 6H-SiC NPs. (d) Schematic illustration of initial laser pyrolysis (ILP) reaction and continuous laser pyrolysis (CLP) reaction mechanisms. (e) TG and (f) DTG curves of pristine PDMS samples at different heating rates. (g) and (h) SEM images of PDMS pyrolysis product samples at heating rates of 10 and 100 °C/min.

The CLP reaction, being the primary process in LPDW technology, can be defined as the heat transfer and diffusion process resulting from the photothermal effect of the moving laser source on the material surface. To gain insight into the CLP reaction mechanism, we conducted an X-ray powder diffraction analysis on the pyrolysis products with varying average power (Figure 2a). The red spectrum exhibited three peaks at 34.9, 35.5, and 38.0°, which clearly indicated the presence of 6H-SiC NPs that were coated at the initial position on the PDMS surface. In contrast, the blue spectrum displayed three peaks at 35.2, 59.6, and 71.7°, which corresponded perfectly to the 3C-SiC product resulting from the CLP reaction. As the laser power was gradually increased from 1.5 to 2.1 W, the peak intensity of the X-ray diffraction (XRD) pattern for the 3C-SiC product intensified due to the higher reaction temperature. These results provide further confirmation that the CLP reaction becomes more pronounced and thorough as the temperature at the center of the laser beam spot increases.

To explore the role of 6H-SiC NPs in laser pyrolysis, we conducted TG analysis on PDMS samples with and without 6H-SiC NPs coating (Figure 2b). The samples, weighing 5 mg and with a mixture ratio of 10:1, were heated under an air atmosphere from 27 to 1700 °C at a rate of 20 °C/min. The weight loss percentage was recorded in the TG curves. Both pristine PDMS (without SiC NPs) and PDMS coated with 6H-SiC NPs (PDMS with SiC NPs) exhibited rapid weight loss at 300 °C, followed by a relatively stable weight loss curve until 600 °C. Specifically, the weight loss of PDMS without SiC NPs decreased from 99.26 to 52.45% at 600 °C, while PDMS with SiC NPs decreased from 99.68 to 50.83% at the same temperature. At 1400 °C, the difference in mass percentage between the two samples was only 2.91%, equivalent to approximately 0.146 mg. Considering the mass difference caused by the presence of 6H-SiC NPs on the PDMS surface, the difference in mass percentage between the two samples is negligible. The corresponding DTG curves revealed that the prominent peaks of the weight loss rate (Derivative) curves for both samples (PDMS with and without SiC NPs) appeared around 400, 460, 500, and 510 °C, respectively (Figure 2c). Although there are some subtle differences in the weight loss rate curves between the two samples from 300 to 600 °C, the overall trend is similar. These results essentially indicate that the 6H-SiC NPs do not participate in the laser pyrolysis reaction of PDMS but rather serve as endothermic sources for inducing the photothermal effect by absorbing laser energy. Figure 2d provides a detailed illustration of the ILP and CLP processes. The ILP reaction is triggered by the absorption of laser energy at the initial point of the 6H-SiC NPs. The resulting laser heating region acts as the pyrolysis center, with heat diffusing downward through the PDMS surface, generating opaque initial and continuous pyrolysis products (3C-SiC) in the conduction heating region. With a slight movement of the laser, the laser heating region overlaps with the new pyrolysis region, causing the conduction heating region to move along with the laser, re-forming a new pyrolysis region and continuous pyrolysis products. Finally, the CLP pyrolysis reaction completes the iteration. Although several experiments and calculations have investigated the pyrolysis mechanism of PDMS, a comprehensive analysis of the pyrolysis process and products is still lacking.^41,51−53^ To address this gap, we summarize the differences between the pyrolysis mechanism of PDMS at low and high heating rates in Figure S8, discussed in the Supporting Information, Discussions 2.4.

**Figure 3:**
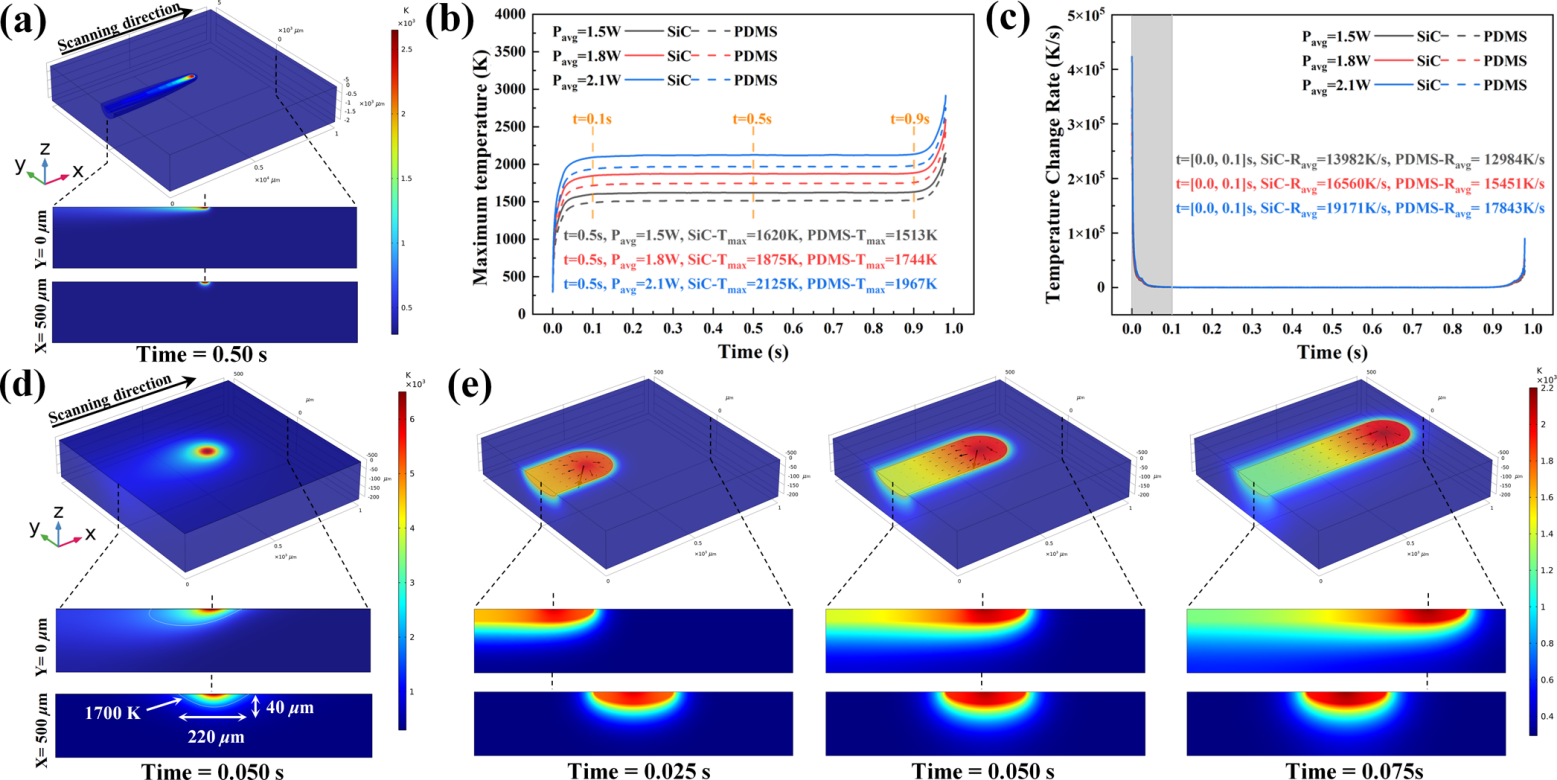
(a) 3D and cross-sectional isothermal surface distributions of PDMS and SiC during CLP reaction (*P*_avg_ = 1.5 W, *v*_ss_ = 10 mm/s, and time = 0.5 s). (b) and (c) Maximum temperature and maximum temperature change rate curves of PDMS and SiC with time under different laser average powers (1.5, 1.8, and 2.1 W). (d) 3D and cross-sectional temperature distributions of PDMS and SiC during ILP reaction (time = 0.050 s). (e) 3D and cross-sectional temperature evolution distributions of the PDMS and SiC during CLP reaction (time = 0.025, 0.050, and 0.075 s).

To further confirm the difference in the pyrolysis mechanism of pristine PDMS at different heating rates, TG and DTG analyses were conducted to investigate the conversion process of PDMS to 3C-SiC under various hearing rates (Figure 3e,f). By comparing the results, we observed that the remaining weights of the three PDMS samples started to decrease at the same temperature of 270 °C, and the weights remained relatively stable at around 560, 720, and 770 °C, respectively. At 1000 °C, the remaining weights of the three samples were 45.64, 53.90, and 58.56%, respectively. The corresponding DTG curves indicated that the three PDMS samples reached the first peak weight loss rate at 364, 439, and 440 °C, respectively. As the temperature increased, the second peak of weight loss rate for the three PDMS samples appeared at 484, 550, and 570 °C, respectively. It is evident from the DTG curve that the peak weight loss rate of the PDMS samples increased with the heating rate, indicating a more intense pyrolysis process. Interestingly, the TG curves revealed that the weight loss of PDMS samples decreased with the heating rate. To explain this phenomenon, we utilized scanning electron microscopy (SEM) to characterize the pyrolysis products of PDMS under heating rates of 10 and 100 °C/min (Figure 2g,h). At a heating rate of 10 °C/min, PDMS undergoes low-heating-rate pyrolysis, leading to the breaking and reformation of Si–O bonds in the PDMS chain, ultimately forming cyclic oligomers. This thermal degradation process results in random cracks within the PDMS, promoting further pyrolysis of cyclic oligomers to 3C-SiC at high temperatures. In contrast, at a heating rate of 100 °C/min, PDMS undergoes high-heating-rate pyrolysis, resulting in the formation of silicon oxides on the PDMS surface through the breaking and re-formation of Si–CH_3_ bonds. These silicon oxides create an oxygen-deficient environment within the PDMS, inhibiting further thermal degradation and direct reduction to 3C-SiC by pyrolytic carbon at high temperatures.

In the laser pyrolysis process, the heating rate is extremely high, far exceeding the critical conditions of the PDMS pyrolysis mechanism. Consequently, considering the pyrolysis mechanism of PDMS, we can infer that PDMS undergoes a direct transformation into 3C-SiC through laser pyrolysis.

### 2.3 Thermal Effect of LPDW Technology

Considering the extreme temperatures involved in the laser pyrolysis process, it becomes challenging to experimentally monitor the heating rate and temperature. To tackle this issue, we employed the finite element method (FEM) to investigate the initial and continuous laser pyrolysis processes based on the previous mechanisms. The physical models and parameter settings are discussed in the Supporting Information, Discussions 2.5.

To validate the heating rate during the CLP process, we constructed a model that aligns with the experimental conditions (10 × 10 × 2 mm). The 3D isothermal surface distribution revealed the temperature distribution and heat transfer of the PDMS substrate and SiC pyrolysis product during the CLP process (Figure 3a). The high temperature was primarily concentrated at the laser spot and gradually decreased in a wave-like gradient along the opposite direction of laser movement. Accordingly, the heat was predominantly concentrated in the SiC pyrolysis product and slightly extended into the PDMS through the interface between SiC and PDMS. Additionally, the cross-sectional isothermal surface distributions illustrated that the new pyrolysis product in the heat conduction region played a significant role in the CLP process. Based on this observation, we extracted the maximum temperature and maximum temperature change rate of the entire simulation model under different laser power and scanning time (Figure 3b,c). It was observed that the maximum temperature curves of PDMS and SiC exhibited three stages over time. Initially, from 0.0 to 0.1 s, the temperature increased rapidly due to laser irradiation. During this stage, heat generation dominates over heat dissipation (heat conduction, heat radiation, and heat convection), leading to the rapid accumulation of heat in the SiC pyrolysis products. In the subsequent stage, from 0.1 to 0.9 s, the maximum temperature curves remained stable. This phenomenon can be attributed to the dynamic balance between heat generation and heat dissipation during the CLP process. In the final stage, from 0.9 and 1.0 s, the maximum temperature curve experienced a sudden rise. This can be attributed to heat losing its primary heat conduction path as the laser moved to the edge of the model.

Furthermore, as the laser power increased from 1.5 to 1.8 and 2.1 W, the maximum temperature of PDMS and SiC rose from 1513 to 1744 and 1967 K, and from 1620 to 1875 and 2125 K at 0.5 s, respectively. It is important to note that during laser pyrolysis, the SiC pyrolysis products conduct most of the heat due to their excellent thermal conductivity. The curve of the highest temperature change rate further supports the temperature trend during the CLP reaction. At a *P*_avg_ of 1.5 W, the average maximum temperature change rates (*R*_avg_) for PDMS and SiC were 12,984 and 13,982 K/s from 0 to 0.1 s, respectively. With the increase in *P*_avg_, the average maximum temperature change rates of PDMS and SiC increased to 15,451 and 16,560 K/s at 1.8 W, and 17843 and 19171 K/s at 2.1 W, respectively. Such a high-temperature change rate exceeds the critical heating rate in the high-heating-rate pyrolysis route of PDMS, further confirming that PDMS is directly converted into 3C-SiC pyrolysis products during the CLP reaction.

To investigate the temperature and temperature change rate during the laser pyrolysis in detail, we further optimized the simulation model to improve the simulation accuracy (1000 × 1000 × 200 μm). Moreover, considering the distinctions between the ILP and CLP processes, we initially examined the 3D and cross-sectional temperature distributions at the intermediate stage of the ILP process (Figure 3d). It was evident that the 6H-SiC layer absorbed the laser energy and reached the maximum temperature of 6409.10 K, leading to heat spreading in all directions. In the section parallel to the scanning direction (*Y* = 0 μm), the temperature distribution within the PDMS substrate closely resembled the shape of a wing section. In the section perpendicular to the scanning direction (*X* = 500 μm), the temperature distribution exhibited a semi-elliptical pattern consistent with the morphology of the PDMS microchannel. Although there are slight discrepancies compared to the experimental results (124 μm and 38 μm in Figure 1c), this temperature can largely serve as a reference for the critical temperature required for PDMS conversion into 3C-SiC. The 3D isothermal surface distributions of the initial pyrolysis process, along with the corresponding top view and cross-sectional views, are illustrated in Figure S11.

The temperature evolution distributions in 3D and cross sections during the CLP process were also investigated (Figure 3e). It was observed that the high-temperature region was concentrated around the 3C-SiC pyrolysis products directly exposed to the laser, with maximum temperatures recorded at different time points: 2054.64, 2137.67, and 2177.76 K. The disparity in heat capacity and thermal conductivity of the PDMS substrate and 3C-SiC pyrolysis products resulted in heat accumulation within the 3C-SiC and a limited amount of heat conduction into the PDMS during the CLP process. In the section parallel to the scanning direction (*Y* = 0 μm), the temperature distribution exhibited significant heat accumulation on the same side as the laser motion, attributed to the conduction limitation of PDMS. Conversely, on the opposite side, the heat was conducted through the 3C-SiC pyrolysis products. The black arrow indicates the thermal conduction direction corresponding to the 3C-SiC pyrolysis product. In the section perpendicular to the scanning direction (*X* = 500 μm), the temperature distribution revealed that the 3C-SiC pyrolysis product primarily absorbed and conducted heat, which was then transferred to the PDMS substrate.

These results align with the roles of the laser heating region and heat conduction region in the previously discussed laser pyrolysis mechanism, providing a clear depiction of the laser pyrolysis process of PDMS. Furthermore, in addition to the heating rate and temperature, the deformation and stress involved in the CLP process are illustrated in Figures S12 and S13 and are further discussed in the Supporting Information, Discussions 2.6.

### 2.4 Design and Fabrication of Flexible Pressure Sensors

**Figure 4:**
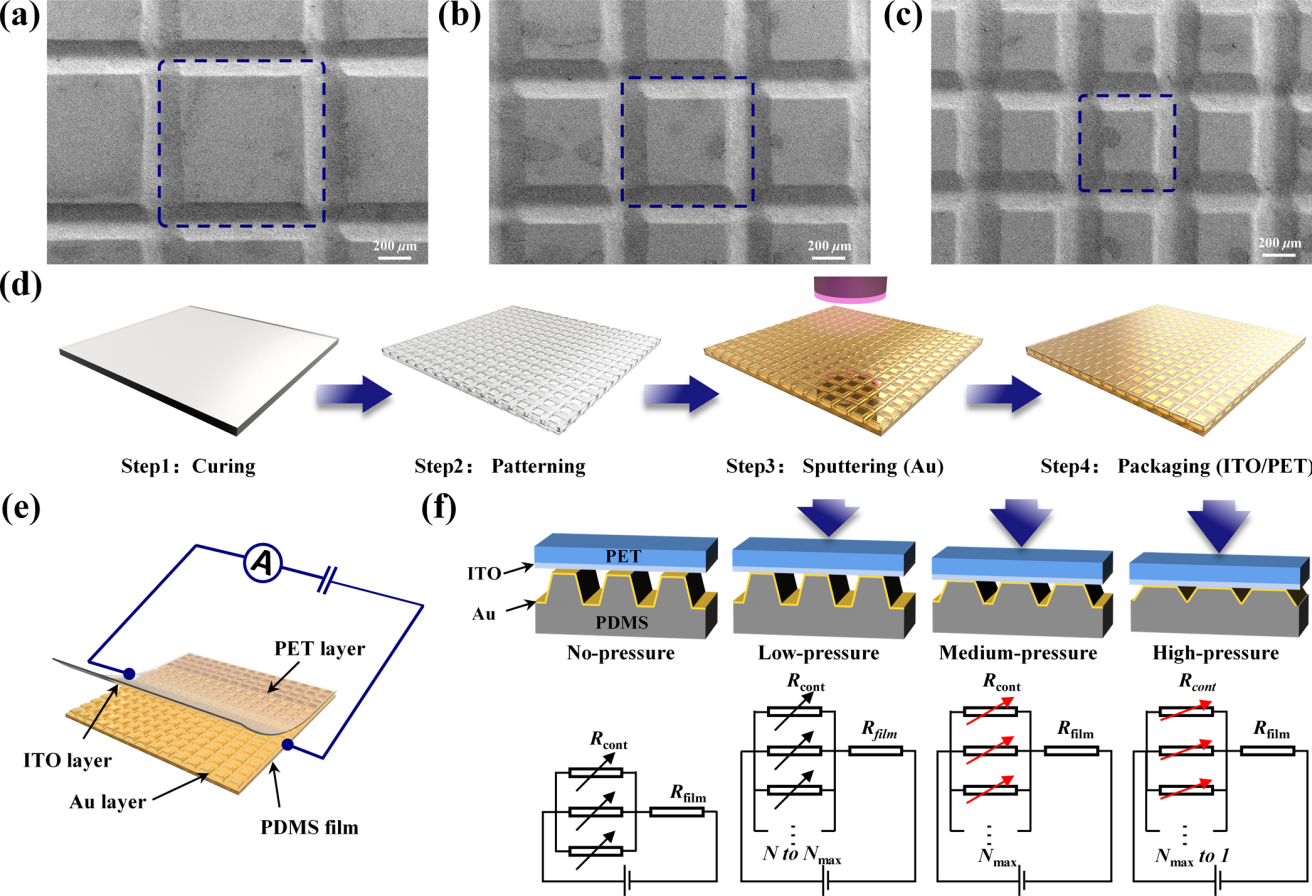
(a)–(c) Surface topography of PDMS micro-truncated pyramid arrays with different scanning pitches (#12 × 12, #16 × 16, and #20 × 20) fabricated using continuous laser direct writing technology. (d) Schematic illustration of the fabrication process for flexible pressure sensors with micro-truncated pyramid array structure. (e) Simplified circuit schematic for testing the performance of the flexible pressure sensor. (f) 3D cross-sectional schematics and equivalent circuit diagrams of the sensor under different pressure conditions.

To assess the potential application of LPDW technology in creating PDMS microarrays, we designed and fabricated flexible pressure sensors featuring truncated pyramid microarray structures of varying pitches. After determining the optimal parameters (*P*_avg_ = 1.5 W, *f*_pr_ = 40 kHz *v*_ss_ = 10 mm/s, *d*_def_ = −6 mm), we conducted laser scanning on a square area (12 × 12 mm) of the PDMS surface at equidistant intervals in both horizontal and vertical directions, forming a number sign (#) pattern. Due to the inherent limitations of the laser spot size, we adjusted the scanning pitch to control the size of the micro-truncated pyramids. The resulting microstructure arrays with different laser scanning pitches (#12 × 12, #16 × 16, and #20 × 20) were characterized using scanning electron microscopy (Figure 4a–c). These results demonstrate remarkable consistency and reproducibility in the geometric shapes of micro-truncated pyramids.

Subsequently, we designed and fabricated flexible pressure sensors incorporating micro-truncated pyramid arrays (Figure 4d). The fabrication process involved mixing a cross-linking agent with the PDMS base, which was then poured into a meticulously flat acrylic template. Following curing, a smooth PDMS film was obtained and patterned using LPDW technology. The PDMS film was subsequently cleaned and subjected to magnetron sputtering with a conductive metal layer (Au). Finally, the PDMS film with a micro-truncated pyramid array was electrically connected to a PET film with an indium tin oxide (ITO) layer using conductive tape and liquid metal and then packaged with heat-shrinkable tubes (refer to the Experimental Method section in the Supporting Information for more details).

To ensure the accurate testing of the flexible pressure sensor, we employed a universal testing machine and a digital source meter to establish a test platform for measuring the sensitivity of the sensors. Specifically, we applied a constant voltage of 1.0 V between the ITO/PET and Au/PDMS electrodes and monitored the current change under various pressure levels. To aid in comprehending the testing process, we provided a simplified circuit model (Figure 4e). The equation for the corresponding mechanism of the sensor can be expressed as follows:
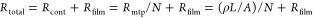
4where the variable *R*_total_ represents the total resistance of the sensor, which consists of resistance *R*_cont_ and film resistance *R*_film_. *R*_cont_ represents the contact resistance between the ITO/PET and Au/PDMS electrodes through the micro-truncated pyramid structures. *R*_film_ represents the additional uncontacted resistance in the ITO/PET and Au/PDMS electrodes, respectively. Accordingly, *R*_cont_ is determined by the contact resistance of an individual micro-truncated pyramid (*R*_mtp_) and the number of parallel connections of micro-truncated pyramid structures (*N*). According to Ohm’s law, ρ is the resistivity of the contact pair between the ITO/PET and Au/PDMS electrodes based on the micro-truncated pyramid structure, *L* represents the thickness of the contact pair, and *A* indicates the contact area of the contact pair.

When external pressure is applied to the flexible pressure sensor, the parameters ρ and *L* are determined by the inherent material properties and remain constant. Therefore, the performance of the piezoresistive pressure sensor, driven by resistance changes, is primarily determined by the parameters *A* and *N*. To enhance the understanding of this mechanism, we presented 3D cross-sectional schematics and equivalent circuit diagrams of the pressure sensor under different pressure conditions (Figure 4f). At low external pressure, the resistance value of the sensor is influenced by the number of contact pairs (*N*) between the electrodes. As external pressure increases, the contact area (*A*) between the electrodes expands, resulting in a change in contact resistance. At high external pressure, adjacent micro-truncated pyramid structures contact each other, reducing the number of contact pairs (*N*) and increasing the contact area (*A*). However, in a parallel circuit, the total resistance is lower than the smallest individual resistances. At this stage, the contact pairs of the micro-truncated pyramid array form a large contact block, and the minimum resistance of the contact block determines the final resistance of the sensor.

### 2.5 Sensing Performance of Flexible Pressure Sensors

To assess the sensing performance of the flexible pressure sensor based on micro-truncated pyramid arrays, we conducted various characterizations on sensors with different laser scanning pitches (#12 × 12, #16 × 16, and #20 × 20). The sensor characteristics can be categorized into three main aspects: sensing characteristics, electrical characteristics, and reliability characteristics.

**Figure 5:**
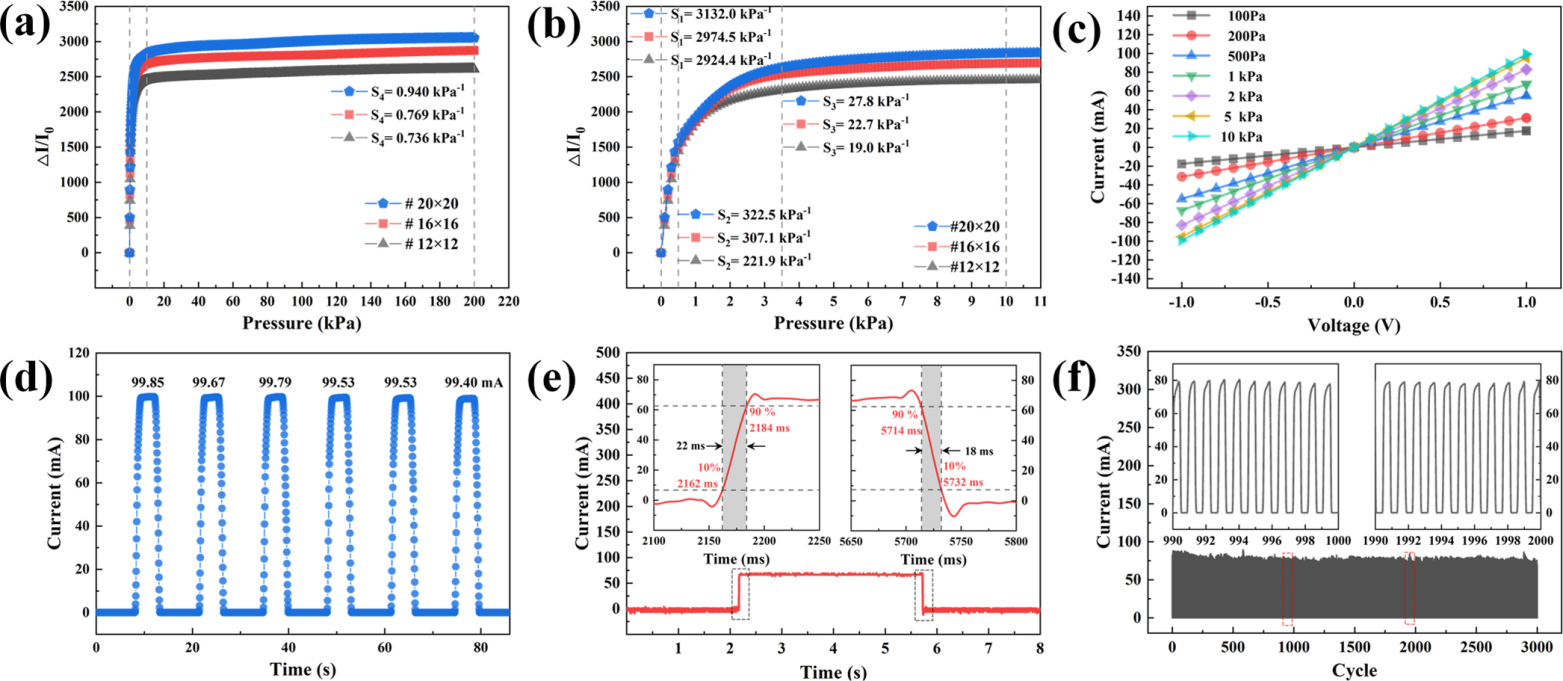
(a) and (b) Sensitivity comparison of pressure sensors with different scanning pitches (#12 × 12, #16 × 16, and #20 × 20). (c) Current–voltage (*I*–*V*) curves of the pressure sensor (#20 × 20) under different external pressures. (d) Current changes in the pressure sensor (#20 × 20) under steady and repeated external pressure (10 kPa). (e) Response time of the pressure sensor (#20 × 20) with an external pressure of 1 kPa. The insets show the response processes of loading and unloading, respectively. (f) Current changes in the pressure sensor (#20 × 20) during 3000 repeated loading/unloading cycles (frequency of about 1.0 Hz, applied pressure of 2 kPa). The insets show the detailed current change curves from 990–1000 to 1990–2000 s.

The sensitivity of the pressure sensor, a crucial sensing characteristic, is determined by the slope of the current change rate curve under different external pressure loads. Accordingly, the sensitivity (*S*) of the piezoresistive sensor is defined as
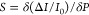
5where *I*_0_ represents the initial current without external pressure, Δ*I* denotes the change in real-time current (*I*) under different pressures, and δ*P* represents the variation of the external pressure.^54^Figure 5a,b illustrates the overall and local relationship between relative current (Δ*I*/*I*_0_) and external pressure (*P*) for three flexible pressure sensors with different scanning pitches. As the laser scanning pitch increased, the saturation current of the device notably increased. This observation aligns with the mechanism described earlier for the pressure sensor. For further studies, we selected the most sensitive device (#20 × 20) as the exemplar. It is evident that the response curves of current to external pressure could be divided into four distinct parts based on their slopes in different pressure ranges. In the pressure range of 0–0.5 kPa, the sensitivity of the sensor was 3132.0 kPa^–1^. In the pressure ranges of 0.5–3.5 and 3.5–10 kPa, the sensitivity was 322.5 and 27.8 kPa^–1^, respectively. In the pressure range of 10–20 kPa, the sensitivity decreased to 0.94 kPa^–1^ while maintaining excellent linearity. These results demonstrate the excellent sensing performance of the pressure sensor at different pressure levels, particularly its high sensitivity in the micro-pressure and low-pressure ranges. Repeatability is also a vital aspect of sensing characteristics. It ensures that the sensor provides a consistent and stable output over time, even under challenging conditions of high pressure and repeated deformation. Figure 5c illustrates the current response curve of the device during the repeatability test. When the device underwent 6 cycles of repeated loading and unloading at 100 kPa, the peak current of the device remained stable at approximately 99.63 mA. The device exhibits a fast and accurate current response, maintaining a consistent peak shape and value for the response current profile across each cycle. This result demonstrates the excellent consistency and repeatability of the device, which is crucial for ensuring accurate sensing in a wide range of applications.

To comprehensively analyze the electrical properties of the flexible pressure sensor, we investigated the current–voltage (*I*–*V*) characteristics and response time characteristics of the device. Figure 5c shows the current–voltage curves of the pressure sensor under different constant external pressure conditions. We observed that the slope of the *I*–*V* curve gradually increased with higher external pressure as the voltage was swept from −1.0 to 1.0 V. Furthermore, the device strictly obeyed Ohm’s law, and each voltage–current curve exhibited exceptional linearity. This result indicates that the device maintains stable electrical characteristics under fixed pressure, which is crucial for its practical application in various scenarios.

Figure 5e illustrates the response time curve of the flexible pressure sensor during loading and unloading. The response time for pressure loading and unloading was defined as the time required for the maximum current to increase from 10 to 90% and decrease from 90 to 10%, respectively.^55^ The sampling frequency was set to 100 kHz, with a 1.0 ms sampling interval. The device exhibited a remarkably rapid response speed during pressure loading and unloading, with response times of 22 and 18 ms, respectively. Although there was a slight overshoot in its response, the sensor maintained excellent dynamic response capability.

To assess the mechanical reliability of the flexible pressure sensor, we subjected the device to 3000 cycles of loading and unloading with an external pressure of 2 kPa and a frequency of approximately 1.0 Hz. Figure 5f illustrates the current output curve of the device during the cycles. While the peak value of the current output curve fluctuated in some cycles, the overall current output remained consistent, indicating the high mechanical stability of the flexible pressure sensor. Even after 1000 and 2000 cycles, the current output of the device maintained excellent stability, demonstrating the reliability of the conductive metal layer on the surface of the micro-truncated pyramid structures.

### 2.6 Potential Applications of Flexible Pressure Sensors

**Figure 6:**
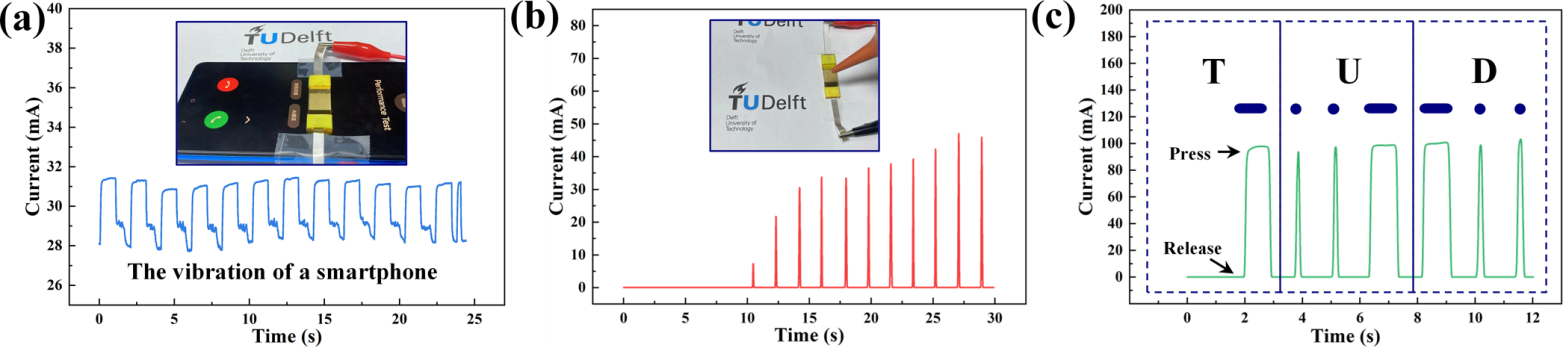
Applications of flexible pressure sensors fabricated by laser pyrolysis direct writing technology: (a) Real-time detection of the vibration of a mobile phone during incoming calls. (b) Real-time detection of the impact of different intensities of airflow by squeezing a rubber suction ball. (c) Real-time detection of Morse code by tapping the three letters of TUD with a finger.

To demonstrate the potential applications of the flexible pressure sensor, we conducted a series of sensor tests that encompassed different pressure ranges. First, we utilized the flexible pressure sensor to detect micro-pressure fluctuations caused by vibrations from a smartphone (Figure 6a). The real-time current response curve of the sensor exhibited precise and periodic changes with the vibration of the smartphone, with the magnitude of the response current corresponding to the intensity of the vibration. This result demonstrates that the flexible pressure sensors can detect not only simple vibrations in acoustic scenarios but also micro-pressure on the surface of various equipment. Furthermore, we employed the flexible pressure sensor to detect real-time responses to airflow (Figure 6b). By adjusting the compression strength of the rubber suction ball, airflow of different intensities was generated to act on the surface of the sensor. The flexible pressure sensor exhibited distinct response peaks for each airflow impact, demonstrating its high-resolution capability in detecting micro-pressure changes. This result indicates the potential application of pressure sensors in high-resolution micro-pressure detection. Additionally, we recorded the real-time response curve while tapping the International Morse code for the letters T, U, and D, where long presses and short taps corresponded to the dash and dot signals, respectively (Figure 6c). This result demonstrates the potential application of pressure sensors in detecting human motion, especially in precise and accurate motion signals.

**Figure 7:**
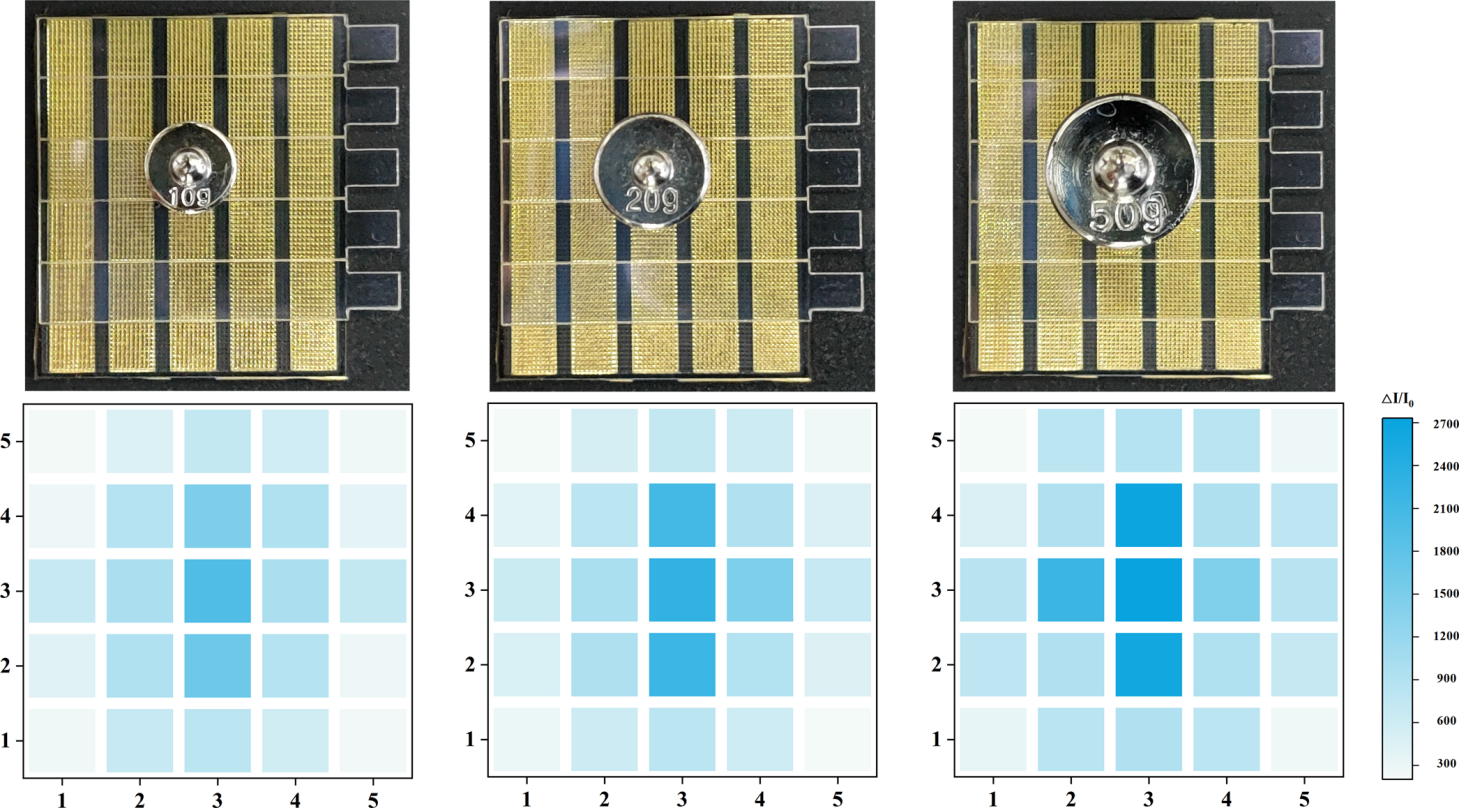
Real-time detection of spatial pressure distribution capabilities on a 5 × 5 sensor array using weights of different masses (10, 20, and 50 g).

To validate the LPDW technology, we successfully fabricated and integrated a proof-of-concept piezoresistive flexible pressure sensor array (5 × 5) consisting of 25 individual sensors based on the micro-truncated pyramid array (Figure 7). To assess its performance, we applied three different mass weights (10 g, 20 g, and 50 g) onto the surface of the sensor array, resulting in varying pressure distributions across the sensor surface.

It was evident that the area beneath the weight appeared darker in color compared to the unloaded region, indicating a significant increase in current flow. Furthermore, as the amount of weight magnitude increased, the color corresponding to the loaded area exhibited a darker shade overall. Moreover, owing to the inherent rigidity of the PET/ITO material, the pressure applied at the center of the weight extended and gradually decreased toward the edges of the sensor array, thereby causing the corresponding area to appear brighter. These results clearly demonstrate the precise spatial pressure detection capability of the integrated pressure sensor array.

## 3 Conclusions

In summary, we have developed a laser pyrolysis direct writing (LPDW) technology for fabricating high-performance flexible pressure sensors with a micro-pyramid array. Through extensive experiments and simulations, we reveal the optimal condition and mechanism for continuous laser pyrolysis (CLP). Using this technology, we designed and fabricated piezoelectric flexible pressure sensors with a micro-truncated pyramid array using conductive PDMS film. The fabricated sensor exhibited remarkable sensitivities of 3132.0, 322.5, and 27.8 kPa^–1^ in the pressure ranges of 0–0.5, 0.5–3.5, and 3.5–10 kPa, respectively. The sensor had a fast response time (loading 22 ms and unloading 18 ms) and demonstrated excellent repeatability and durability over 3000 cycles. Furthermore, the sensor could detect micro and low pressures such as mobile phone vibrations, airflow impacts, and finger touches. It could also be easily integrated into sensor arrays to map the spatial distribution of nonuniform pressure fields. In addition to its outstanding performance, the flexible pressure sensor with a micro-truncated pyramid array fabricated using LPDW technology exhibited the advantages of low cost, high scalability, and easy fabrication. Compared with traditional complex micro/nanofabrication processes, this study has provided a new route for designing and fabricating high-performance flexible pressure sensors.

## Supporting Information Available

Experimental methods include the experimental materials, processes, and equipment; realization conditions; mechanism analysis; and thermal and mechanical effects of laser pyrolysis direct writing technology (PDF)
Demonstrating the occurrence or absence of the CLP reaction (MP4)
