# Supplementary material for: Rapid Fabrication of High-Performance Flexible Pressure Sensors Using Laser Pyrolysis Direct Writing
Source: ACS Appl Mater Interfaces. 2023 Jul 31;15(34):41055–66. doi: 10.1021/acsami.3c04290 (PMC10472334; doi:10.1021/acsami.3c04290)
Supplement: Supplementary file 1 — am3c04290_si_001.pdf [file am3c04290_si_001.pdf]

Supporting Information

Rapid Fabrication of High-Performance Flexible Pressure Sensors Using Laser Pyrolysis Direct Writing

Shaogang Wang[†,‡,∞], *Qihang Zong*[‡,∞], *Huiru Yang*[‡], *Chunjian Tan*[†,‡], *Qianming Huang*[‡], *Xu Liu*[†,‡], *Guoqi Zhang*[†], *Paddy French*[†], *and Huaiyu Ye*[†‡*]

[†]Faculty of EEMCS, Delft University of Technology, Mekelweg 4, 2628 CD Delft, The Netherlands.

[‡]School of Microelectronics, Southern University of Science and Technology, Shenzhen 518055, China.

[∞] These authors contributed equally.

[*] E-mail: hy.ye@tudelft.nl (Huaiyu Ye)

Table of Contents

Index	*Page*
**1. Experimental Methods**	S4
**1.1 Fabrication of the PDMS Film**	S4
**1.2 Fabrication of the Microstructure Arrays**	S4
**1.3 Packaging of the Flexible Pressure Sensors**	S5
**1.4 Characterization and Measurement**	S5
**2. Supplementary Discussion**	S6
**2.1 Realization Conditions of LPDW Technology.**	S6
**Figure S1.** Schematic illustration of continuous laser pyrolysis surface reaction morphology at different pulse repletion frequencies (low and high frequency).	S6
**Supplementary Discussion 2.1**	S6
**Table S1.** The average width and depth of the microchannel with different average power at fixed pulse repetition frequency.	S7
**Figure S2.** 3D laser confocal images of microchannel structures on PDMS surface at different focal distances.	S7
**Figure S3.** Schematic illustration of the relationship between the laser average power (P_{avg}), laser peak power (P_{peak}) and pulse repetition frequency (f_{pr}).	S8
**Figure S4.** The detailed heat map of critical realization conditions for continuous laser pyrolysis (CLP) reaction.	S8
**Supplementary Discussion 2.2**	S8
**Figure S5.** The corresponding scanning electron microscopy (SEM) images of the surface topography under the different numbers of laser scans (N1, N2, and N3).	S9
**Figure S6.** Roughness results of PDMS microchannel sidewall under different laser scanning times (N1, N2, and N3).	S10
**Supplementary Discussion 2.3**	S10
**Figure S7.** Optical, DIC, and 3D topological figures of PDMS microchannels under different numbers of laser scans (N1, N2, and N3).	S11
**2.2 Mechanism Analysis of LPDW Technology.**	S11
**Figure S8.** The pyrolysis **mechanisms** of PDMS under low-heating-rate pyrolysis and high-heating-rate pyrolysis routes.	S11
**Supplementary Discussion 2.4**	S11
**2.3 Thermal and Mechanical Effects of LPDW Technology**	S13
**Figure S9.** Schematic **illustration** of laser pyrolysis parameters in the defocused condition.	S13
**Figure S10.** The functional **relationship** between the spot radius ($\omega(z)$) and the defocus distance (d_{Def}) in the defocused state.	S13
**Supplementary Discussion 2.5**	S14
**Figure S11.** (a) 3D **isothermal** surface distribution of the initial pyrolysis process and corresponding top view (b) and cross-sectional views (c) and (d) (Y = 0 μm and X = 500 μm).	S17

**Figure S12.** 3D and cross-sectional distributions of the deformation evolution of PDMS and SiC during continuous laser pyrolysis (Time = 0.025, 0.050, and 0.075 s).	S17
**Figure S13.** 3D and cross-sectional distributions of the stress evolution of PDMS and SiC during continuous laser pyrolysis (Time = 0.025, 0.050, and 0.075 s).	S18
**Supplementary Discussion 2.6**	S18
**Figure S14.** The principal stress line distributions of PDMS and SiC during continuous laser pyrolysis (Time = 0.025, 0.050, and 0.075 s).	S20
**3. Supplementary References**	S20

1. Experimental Methods

1.1 Fabrication of the PDMS Film

The liquid mixture of PDMS precursors (Sylgard 184, Dow Corning, mixture ratio: 10:1) was poured onto a smooth plastic template and placed inside a vacuum chamber for degassing. The mixture was then cured at 80 °C for 2 hours to form a 2 mm thick transparent PDMS film.

1.2 Fabrication of the Microstructure Arrays

The micro-truncated pyramid arrays were fabricated using laser pyrolysis direct writing technology on the PDMS surface. This was achieved by using an ultraviolet (UV) pulsed laser system (Grace X 355-3A, Han's Laser Technology Industry Group Co., Ltd., wavelength: 355 nm) operating at a fixed scanning speed (v_{ss}) of 10 mm/s, a laser average power (P_{avg}) of 1.5 W, and a pulse repetition frequency (f_{pr}) of 40 kHz. SiC nanoparticles (Bide Pharmatech Co., Ltd., particle size: 0.5-0.7 μm) were coated at the initial point of the scan path to induce the continuous laser pyrolysis reaction. After fabrication, the pyrolysis products (3C-SiC) on the PDMS surface were cleaned with an ultrasonic cleaner using anhydrous ethanol and dried with nitrogen gas. A 150 nm thick gold conductive layer was deposited onto the PDMS film (12 × 12 mm) with micro-truncated pyramid arrays using magnetron sputtering equipment (KT-Z1650PVD, Zhengzhou Ketan Instrument Equipment Co., Ltd.)

1.3 Packaging of the Flexible Pressure Sensors

The commercial single-sided conductive tapes (3M7766-50, 3M Company) were utilized as lead wires to connect the ITO/PET electrode (South China Xiangcheng Technology Co., Ltd, thickness: 125 μm, resistance: ≤10 Ω/sq) and Au/PDMS electrode, respectively. Liquid metal (Alfa Aesar Chemical Co., Ltd., metal basis: 99.99 % Ga-In-Sn) was coated on the conductive tapes. Subsequently, the ITO/PET electrode and Au/PDMS electrode were aligned and assembled by heating a heat-shrinkable tube at 100 °C to complete the final packaging of the flexible pressure sensor.

1.4 Characterization and Measurement

The morphology of the PDMS film with micro-truncated pyramid arrays was characterized using a scanning electron microscope (SEM, Gemini 300, ZEISS) and a 3D laser scanning microscope (VK-X1000, KEYENCE). Thermal gravimetric analysis (TGA, STA 449C, NETZSCH) was employed to investigate the changes in gravimetric components during the pyrolysis of PDMS. The samples were heated from 27 °C to 1400 °C at a ramp rate of 20 °C/min in air. The pyrolysis products were characterized using X-ray diffraction (XRD, Rigaku Smartlab) with a scanning rate of 10°/min. The mechanical performance of the flexible pressure sensor was evaluated using a universal testing machine (TSE503A, Wance Testing Machine Co., Ltd.). The corresponding electrical signals were measured using a digital source meter (2450, Keithley), and the response time was recorded using an oscilloscope (TBS2104B, Tektronix).

2. Supplementary Discussions

2.1 Realization Conditions of LPDW Technology

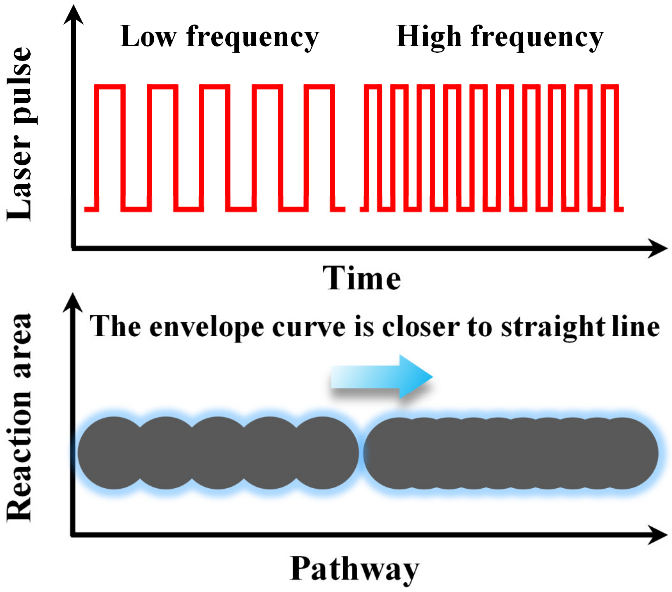

Figure S1. Schematic illustration of continuous laser pyrolysis surface reaction morphology at different pulse repletion frequencies (low and high frequency).

Supplementary Discussion 2.1

In detail, the laser beam acts as an electromagnetic wave that carries both energy and momentum. When it interacts with the PDMS surface and 6H-SiC NPs, it not only provides energy to the 6H-SiC NPs at the initial position through the photothermal effect but also generates radiation pressure on the PDMS surface. The high photon density of the laser causes the optical pressure at the focal plane to be significantly higher than the natural optical pressure of 0.5 dyne[1]. Furthermore, radiation pressure causes the state of 6H-SiC on the PDMS surface to be unstable, making it difficult to accumulate heat and trigger continuous laser pyrolysis

reactions.

Table S1. The average width and depth of the microchannel with different average power at fixed pulse repetition frequency.

Conditions	Average Depth	Average Width	Aspect ratio
P_{avg}= 1.50W f_{pr}=30 kHz	39.2 μm	258.1 μm	0.152
P_{avg}= 1.35W f_{pr}=30 kHz	37.0 μm	237.5 μm	0.155
P_{avg}= 1.20W f_{pr}=30 kHz	33.8 μm	226.1 μm	0.149
P_{avg}= 1.05W f_{pr}=30 kHz	31.0 μm	205.6 μm	0.151
P_{avg}= 0.90W f_{pr}=30 kHz	28.7 μm	192.5 μm	0.149

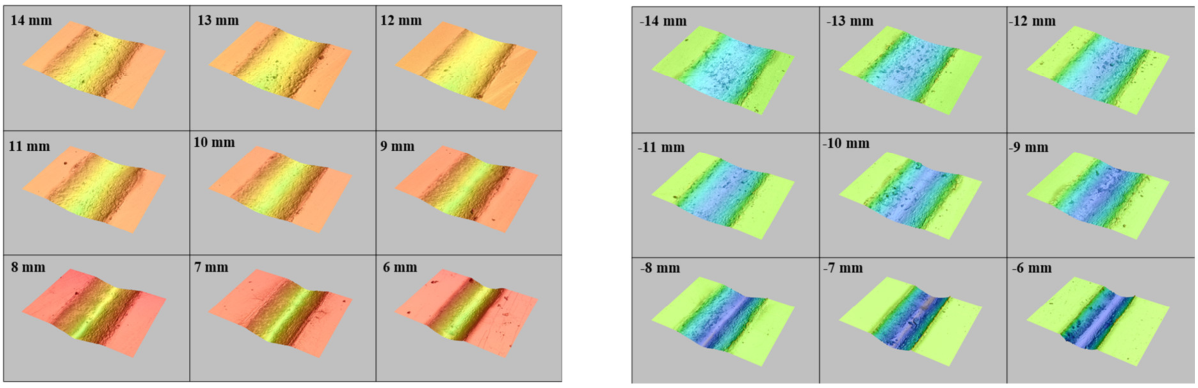

Figure S2. 3D laser confocal images of microchannel structures on PDMS surface at different focal distances.

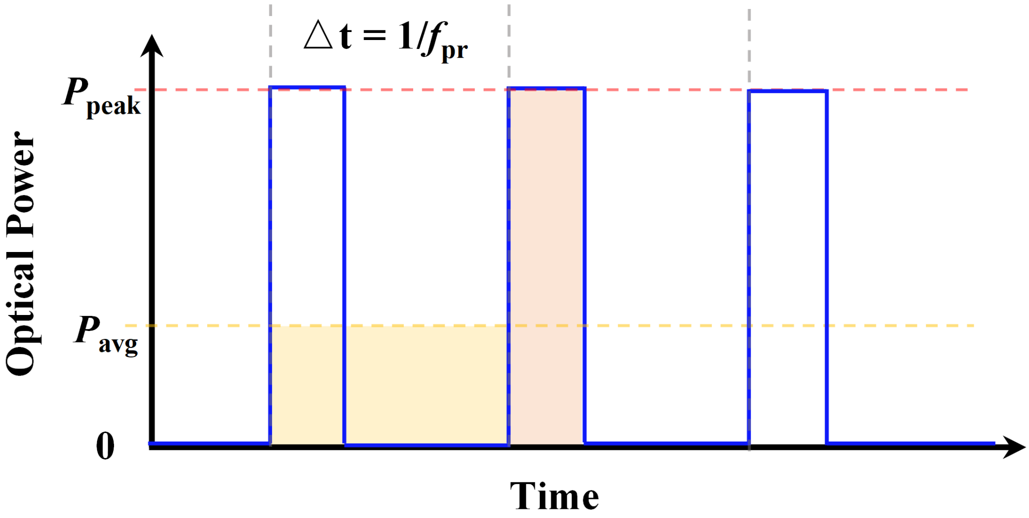

Figure S3. Schematic illustration of the relationship between the laser average power (P_{avg}), laser peak power (P_{peak}) and pulse repetition frequency (f_{pr}).

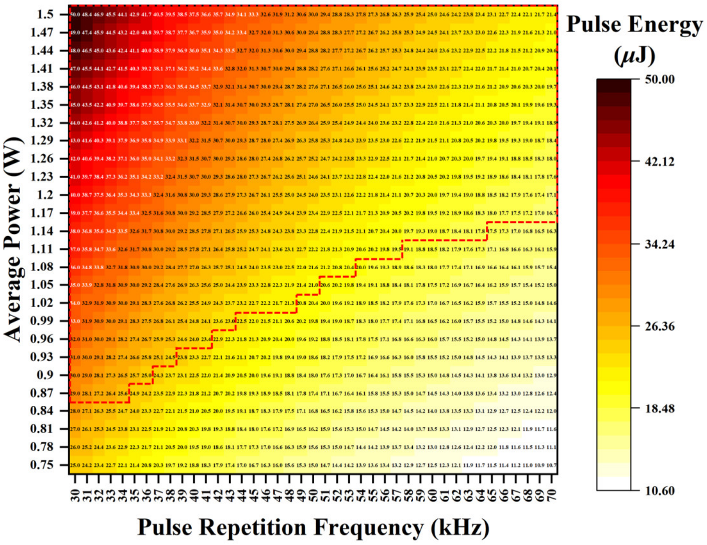

Figure S4. The detailed heat map of critical realization conditions for continuous laser pyrolysis (CLP) reaction.

Supplementary Discussion 2.2

In detail, increasing the average power (P_{avg}) of the laser at the fixed repetition frequency (f_{pr}) and scanning speed (v_{ss}) did not effectively improve the aspect ratio of the microchannels, which remained stable at around 1.55, as shown in **Table S1**. On the other hand, the width and depth of the microchannels increased simultaneously with the laser power. It is worth noting

that the final morphology of the microchannel is jointly determined by the critical pyrolysis temperature of continuous laser pyrolysis and the morphology of SiC pyrolysis products. The overall temperature during laser pyrolysis significantly increases as the laser power increases. However, heat conduction in the medium remains isotropic[2]. The temperature distribution in the system formed by the PDMS substrate and SiC pyrolysis products still follows the Gaussian temperature distribution. As the laser power increases, the temperature of the laser-irradiated SiC pyrolysis product also rises. Due to the constant thermal conductivity of the SiC pyrolysis product, its overall temperature increases and maintains isotropic conduction. However, the critical temperature for laser pyrolysis remains unchanged. Therefore, while the laser moves, the cross-section of the PDMS microchannel expands uniformly in all directions. Consequently, increasing the laser power leads to an increase in the depth and width of the SiC pyrolysis product morphology, which also keeps the aspect ratio of the microchannel unchanged. In contrast, increasing the number of laser scans gradually increases the etching depth of laser scanning due to the high temperature and stress generated during the pyrolysis process, as shown in **Figure S5**. As the etching depth increases, the depth of the PDMS microchannels after laser pyrolysis increases, which leads to an increase in the aspect ratio of the microchannels.

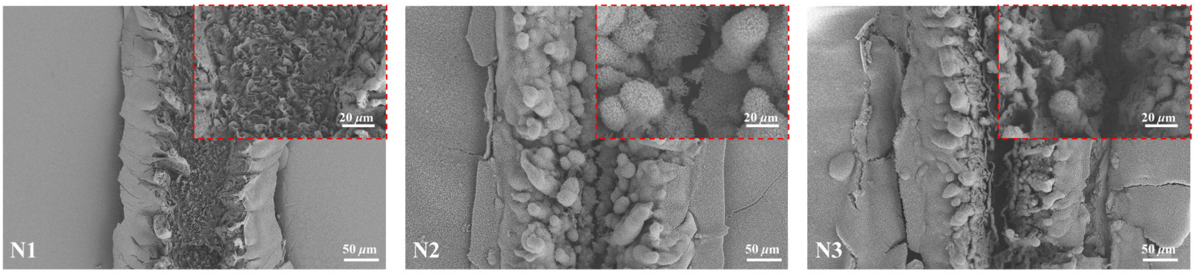

Figure S5. The corresponding scanning electron microscopy (SEM) images of the surface topography under the different numbers of laser scans (N1, N2, and N3).

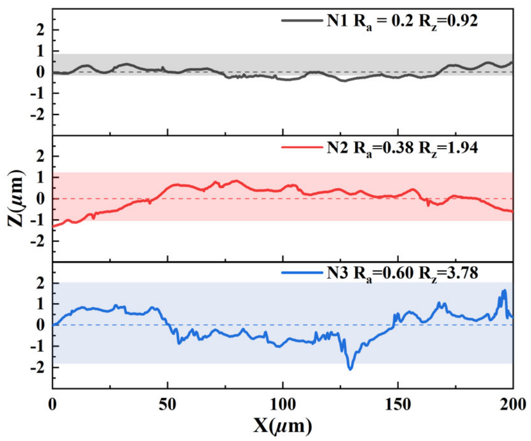

Figure S6. Roughness results of PDMS microchannel sidewall under different laser scanning times (N1, N2, and N3).

Supplementary Discussion 2.3

In detail, **Figure S6** and **Figure S7** show the roughness results and 3D laser confocal images of the microchannel sidewalls with different numbers of laser scans. To avoid measurement errors resulting from the groove-like shape of the microchannel cross-section, line roughness analysis was conducted on the roughest position of the microchannel sidewall for varying numbers of laser scans. The average roughness (R_a) of the microchannel sidewall increased from 0.2 μm to 0.38 μm and 0.60 μm with an increase in the number of scanning times. Meanwhile, the average roughness depth (R_z) also increased from 0.92 μm to 1.94 μm and 3.78 μm, respectively. These results clearly show that the microchannel roughness increases with multiple laser scans compared to a single laser scan. This is because as the number of laser scans increases, the 3C-SiC pyrolysis product extends further into the PDMS, leading to an increase in thermal expansion and interfacial stress between the PDMS substrate and the 3C-SiC pyrolysis product. As a result, the interlayer dislocation between the 3C-SiC pyrolysis products and PDMS results in an increase in the surface roughness of the PDMS microchannels.

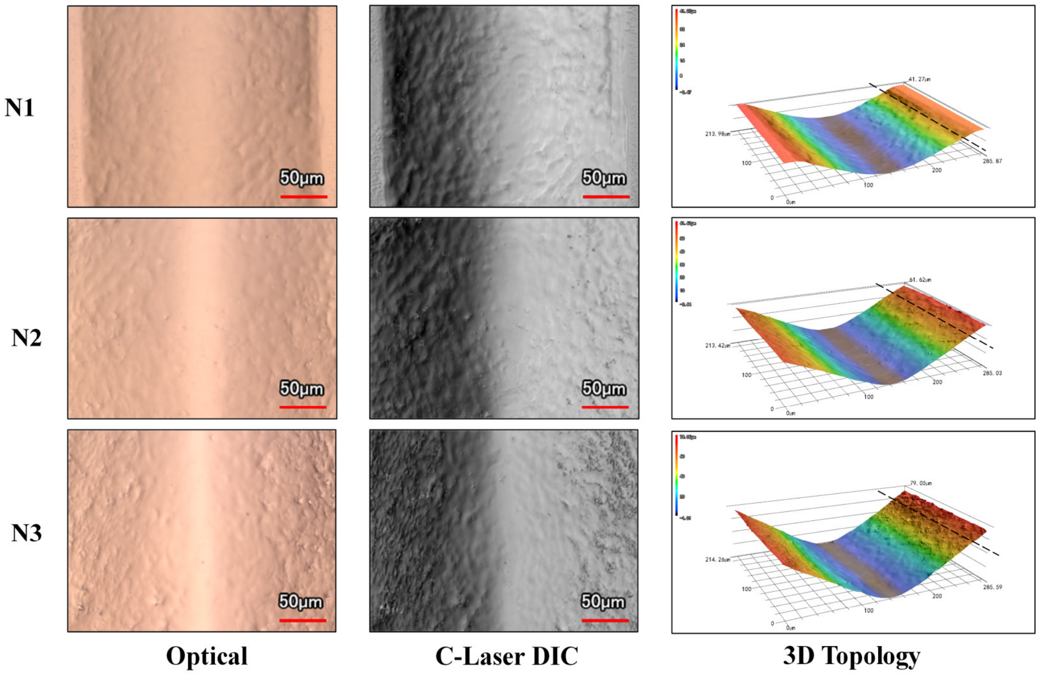

Figure S7. Optical, DIC, and 3D topological figures of PDMS microchannels under different numbers of laser scans (N1, N2, and N3).

2.2 Mechanism Analysis of LPDW Technology

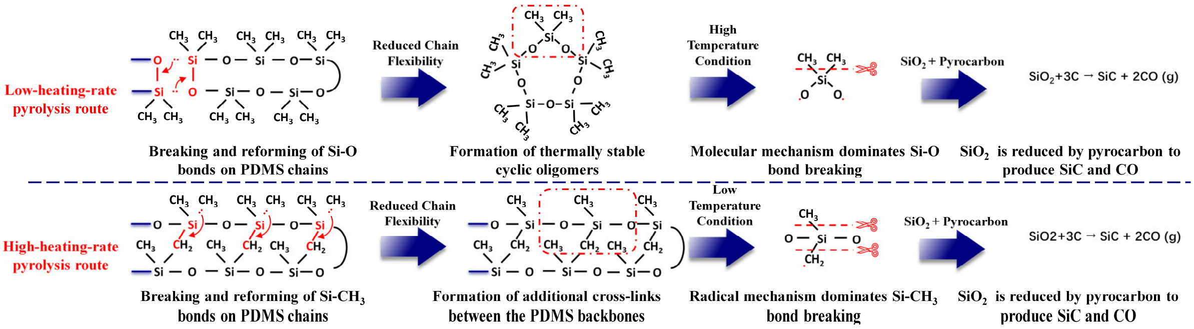

Figure S8. The pyrolysis mechanisms of PDMS under low-heating-rate pyrolysis and high-heating-rate pyrolysis routes.

Supplementary Discussion 2.4

The schematic diagram of the pyrolysis mechanism under high and low heating rate conditions is illustrated in **Figure S8**. In fact, the conversion mechanism of PDMS to 3C-SiC

through continuous laser pyrolysis is influenced by various physical conditions. Burn et al. reported on the conversion of siloxane polymers to silicon carbide, which provided a general theory for the pyrolysis of polysiloxane-based materials through a two-step successive pyrolysis[3]. Although their research mainly focuses on siloxane polymers rather than PDMS, it is still informative. Subsequently, Camino et al. conducted further investigations on the pyrolysis of PDMS through experiments and simulations, which revealed two competing mechanisms of molecular (Low heating rate pyrolysis route) and radical mechanisms (High heating rate pyrolysis route) in the pyrolysis behavior of PDMS at different heating rates[4, 5]. Based on the above, Shin et al. proposed a reaction path for laser pyrolysis of PDMS that directly converted it into SiC at low temperature[6].

However, the comparative studies on laser pyrolysis products and processes still need to be clarified. Therefore, we compared and summarized the low-heating-rate and high-heating-rate pyrolysis routes. When the heating rate is lower than 50 °C/min, PDMS is pyrolyzed into various cyclic oligomers at around 500 °C. In this phase, the molecular mechanism dominates and causes the breaking and reforming of Si-O bonds on PDMS chains to form cyclic oligomers. This further induces tight cross-linking of PDMS chains, leading to an increase in thermal stability. Subsequently, under conditions of low chain flexibility, the cyclic oligomers decompose into SiC with a further increase in temperature. When the heating rate is higher than 100 °C/min, PDMS is pyrolyzed into more stable carbide or oxycarbide of silicon in the low-temperature range of 600 °C to 700 °C. The radical mechanism dominates and inhibits the molecular mechanism in this phase, inducing the breaking and reforming of $Si-CH_3$ bonds on PDMS chains to increase the additional cross-linking of PDMS further. This mechanism also

leads to the condensation of the remaining main chains, resulting in the derivation of silicon oxides. Finally, the oxides of silicon are reduced by the pyrocarbon in the absence of oxygen to produce silicon carbide (SiC) and carbon monoxide (CO).

2.3 Thermal and Mechanical Effects LPDW Technology

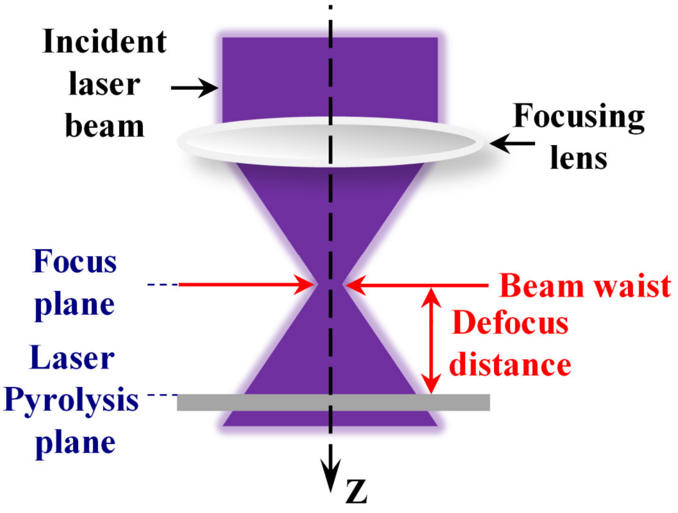

Figure S9. Schematic illustration of laser pyrolysis parameters in the defocused condition.

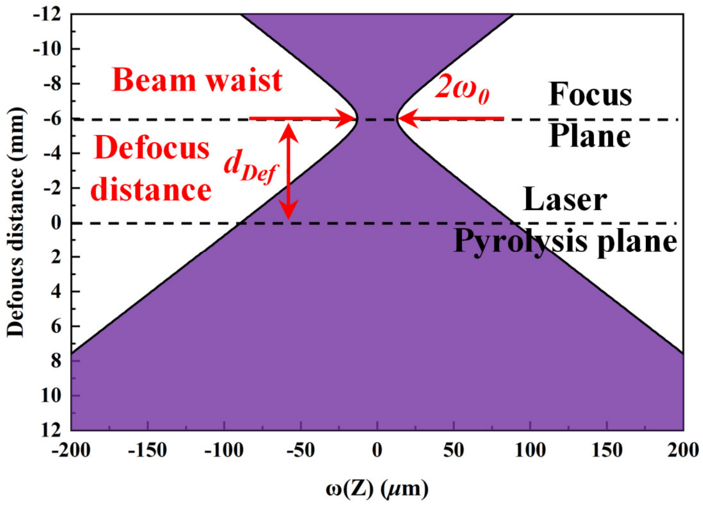

Figure S10. The functional relationship between the spot radius ($\omega(z)$) and the defocus distance (d_{Def}) in the defocused state.

Supplementary Discussion 2.5

During laser scribing, the pyrolysis reaction occurs in a time sequence. Above all, the 6H-SiC NPs at the initial point induce the pyrolysis of PDMS via the photothermal effect, resulting in the generation of 3C-SiC products. Subsequently, the 3C-SiC pyrolysis product absorbs the laser energy and induces the continuous pyrolysis reaction of PDMS in laser moving direction. Consequently, the pyrolysis reaction can be categorized into two different models: the continuous laser pyrolysis process and the initial laser pyrolysis process.

After making some trade-offs between calculation accuracy and calculation time, certain assumptions were applied to FEM (Finite Element Method) calculations, as follows:

(1) The UV laser beam was assumed to follow the Gaussian distribution and was vertically incident on the surface of the SiC pyrolysis product.

(2) The surface morphology of SiC pyrolysis products was assumed to be flat.

(3) The absorption of laser energy on the surface of SiC pyrolysis products was assumed to follow the Beer-Lambert law and was considered a volume heat source.

(4) The heat transfer direction between SiC pyrolysis products and PDMS was assumed to be isotropic.

(5) The heat loss due to phase transition during laser pyrolysis was assumed to be negligible.

After considering the reflection loss and absorption coefficient of the Gaussian light source in the application of the Beer-Lambert law, the heat source density per unit volume at a position

(x, y, z) transformed by the photothermal effect of the UV laser can be expressed as follows in the Spatial Cartesian coordinate system:

$$q(x,y,z) = \alpha(1-R)\frac{2P_{\mathrm{avg}}}{\pi{\omega_{\mathrm{def}}}^2}exp\left[\frac{-2(x^2+y^2)}{\omega(z)^2}\right]exp(-\alpha z) \tag{S1}$$

$$\alpha = \frac{4\pi\kappa}{\lambda_0} \tag{S2}$$

$$R = \frac{(n-1)^2+\kappa^2}{(n+1)^2+\kappa^2} \tag{S3}$$

$$\omega(z) = \omega_0\sqrt{1+\left(\frac{z+d_{\mathrm{Def}}}{z_R}\right)^2} \tag{S4}$$

$$z_{\mathrm{R}} = \frac{\pi{\omega_0}^2}{\lambda_0} \tag{S5}$$

Where the parameters of α and R are absorption and reflectivity coefficients of material at the incident wavelength, respectively[7, 8]. These parameters can be calculated according to the complex refractive index in formulas (S2) and (S3). Furthermore, P_{avg} and ω_{def} denote the average power of the incident laser and the beam radius at the defocused plane, respectively[9].

In the equation (S4) and (S5), $\omega(z)$ is a function of the spot radius size along the direction of beam incidence at different positions, while ω_0 is the beam waist of the incident laser beam. The parameter z_R represents the distance along the incident direction of the beam from the waist to a section where the cross-sectional area is twice that of the waist[10].

Meanwhile, the parameter d_{Def} is defined as the vertical distance between the focal plane and the laser pyrolysis plane in the defocused state, which is consistent with the definition above, as shown in **Figure S9**. The value of d_{Def} can be set to zero (when the laser pyrolysis

plane coincides with the focal plane), positive (when the laser pyrolysis plane is above the focal plane), or negative (when the laser pyrolysis plane is below the focal plane). When d_{Def} is set to -6.0 mm, the corresponding function curve is shown in **Figure S10**. Finally, $exp(-\alpha z)$ is the relative intensity given by the Beer-Lambert law at the different positions inside the 3C-SiC pyrolysis product[11].

As the incident laser energy is absorbed and partially converted into heat, transient heat equation for the spatial distribution$(x, y, z)$of temperature $T(x, y, z, t)$ at time t as define-difference form:

$$\rho C_P \frac{\partial T}{\partial t} - \nabla \cdot k\nabla T = q(x, y, z) \tag{S6}$$

the corresponding initial condition is written as:

$$T(x, y, z, t = 0) = T_{\mathrm{ext}} = 293.15\ \mathrm{K} \tag{S7}$$

the boundary conditions are formulated as:

$$\begin{cases} \vec{n} \cdot \vec{q} = h(T_{\mathrm{ext}} - T) \\ \vec{n} \cdot \vec{q} = \varepsilon\sigma(T_{\mathrm{amb}}^4 - T^4) \\ \vec{n} \cdot \vec{q} = 0 \end{cases} \tag{S8}$$

where the ρ, C_{P}, and k are density, heat capacity, and thermal conductivity of the material in the heat conduction system, respectively. T_{ext} and T_{amb} are the external temperature and ambient temperature, respectively, in the equation (S7) and (S8). Additionally, h, ε, and σ are the heat transfer coefficient, emissivity, and Boltzmann constant[12, 13]. The heat transfer coefficients for the top and bottom surfaces of the material are determined using empirical formulas, resulting in values of 17.9 W/m^2/K and 9.0 W/m^2/K, respectively[14]. These

coefficients are used to characterize the heat exchange process between the material and the surrounding air during natural convection process.

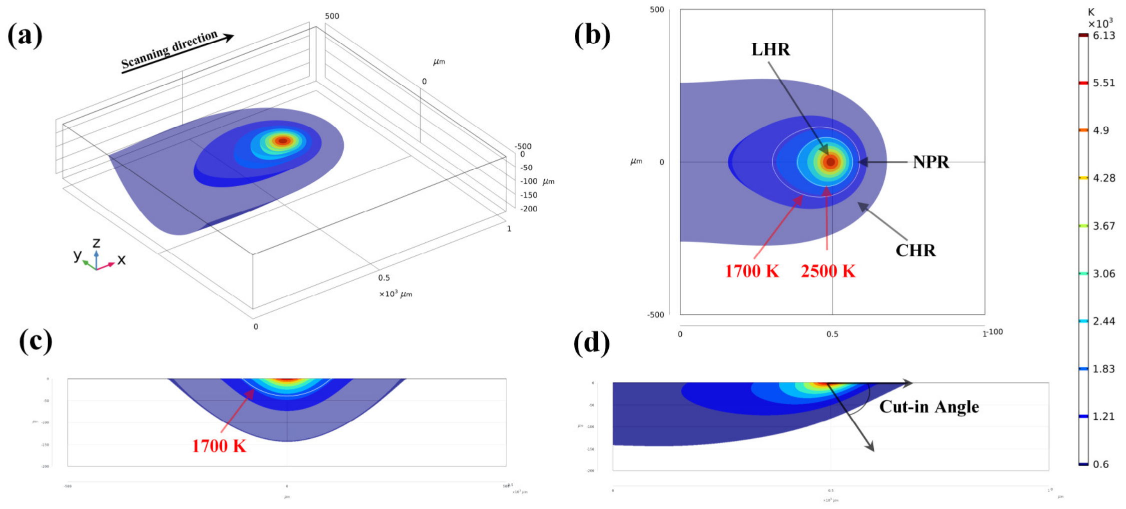

Figure S11. (a) 3D isothermal surface distribution of the initial pyrolysis process and corresponding top view (b) and cross-sectional views (c) and (d) (Y = 0 μm and X = 500 μm).

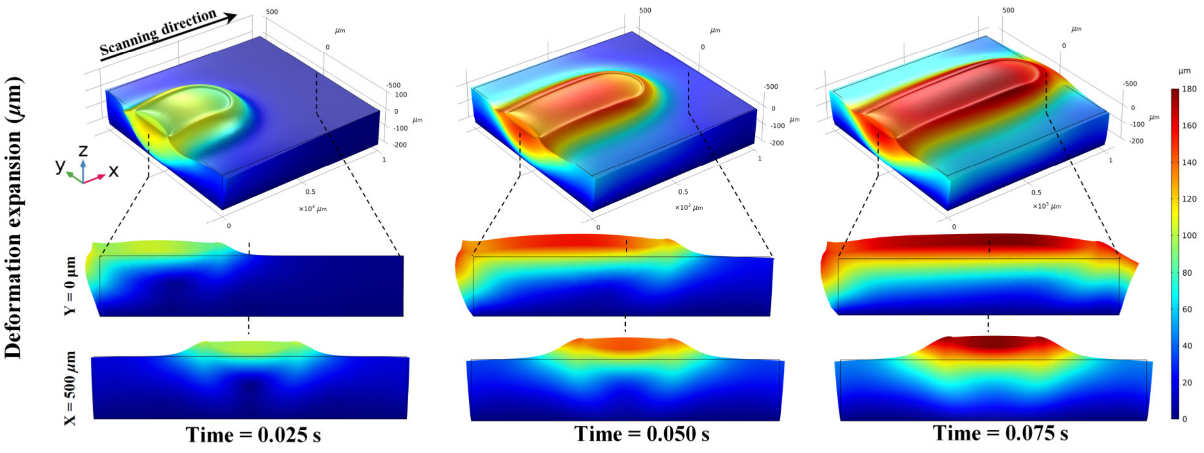

Figure S12. 3D and cross-sectional distributions of the deformation evolution of PDMS and SiC during continuous laser pyrolysis (Time = 0.025, 0.050, and 0.075 s).

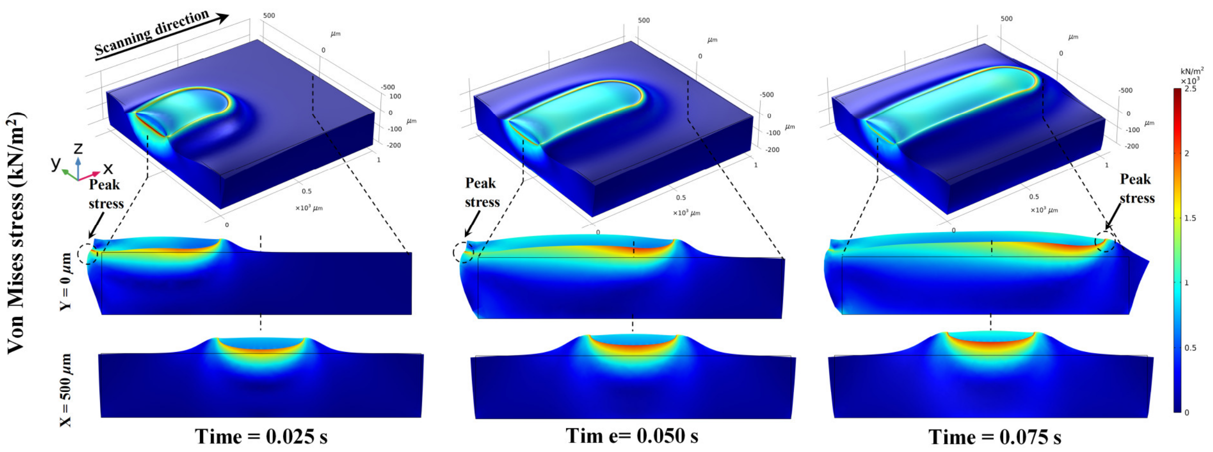

Figure S13. 3D and cross-sectional distributions of the stress evolution of PDMS and SiC during continuous laser pyrolysis (Time = 0.025, 0.050, and 0.075 s).

Supplementary Discussion 2.6

In addition, much attention has been paid to investigating the deformation and stress resulting from the photothermal effect during continuous laser pyrolysis. Therefore, we conducted a thermomechanical coupling analysis of continuous laser pyrolysis. To simulate changes in stress and deformation using the finite element method (FEM), certain assumptions were applied in the computational model, as follows:

(1) The mechanical properties, stress, and strain of the material changed linearly during a small-time increment.

(2) In the plastic zone, the material followed the hardening rule and flow rule.

(3) The material underwent plastic deformation following the law of constant volume.

(4) The yield deformation process of the material followed the Von Mises yield criterion.

When implementing thermomechanical coupling using finite element simulation, it is necessary to analyze and define the elastic-plastic deformation of materials induced by thermodynamics. The elastic strain is determined according to isotropic Hooke's law with temperature-dependent Young's modulus E and Poisson's ratio ν. The thermal strain is obtained from the coefficient of thermal expansion α. Plastic strains are obtained through elastoplastic constitutive equations, which consider the von Mises yield criterion, isotropic hardening rules, and temperature-dependent mechanical properties. The relationship between stress and strain can be expressed as follows

$$\{d\sigma\} = [D]\{d\varepsilon\} - \{C\}dT \tag{S9}$$

where $[D]$ is elastic-plastic stiffness matrix, $\{C\}$ is vector matrix related to temperature T, dT is temperature increment[15].

Figure S12 shows the 3D and cross-sectional distributions of the deformation evolution during continuous laser pyrolysis. It was evident that both the 3C-SiC and PDMS underwent significant thermal expansion due to the laser photothermal effect, and their morphology was consistent with experimental results. Due to the different coefficients of thermal expansion, there was an apparent interlayer mismatch between the 3C-SiC and PDMS. The maximum deformation values at different time stages were 117.0 μm, 155.3 μm, and 180.0 μm, respectively. Interestingly, the maximum deformation occurred behind the position of the laser spot, as indicated by the 2D deformation distribution parallel to the laser scanning direction. This phenomenon is because the deformation of the 3C-SiC pyrolysis product at the center of the spot is limited by the half-surrounding PDMS in the forward direction.

Figure S13 shows the 3D and cross-sectional distributions of the deformation evolution during continuous laser pyrolysis. Obviously, the stress distribution at the interface of PDMS and 3C-SiC was much larger than that in other regions. Meanwhile, the 2D stress distribution parallel to the laser scanning direction showed that the maximum stress positions occurred in the edge region of the PDMS substrate. Correspondingly, the maximum stress values at different time stages were 2213.4 kN/m^2, 1675.2 kN/m^2, and 2466.92 kN/m^2, respectively. This also indicated that the stress distribution in the intermediate stage was more uniform and stable than in the initial and final stages, during the continuous laser pyrolysis process. The two-dimensional stress distribution perpendicular to the laser scanning direction demonstrated that the stress distribution was most concentrated in the area directly below the SiC. This further explains why the SiC pyrolysis products can be easily peeled off from the PDMS substrate. The principal stress line distribution can more intuitively display the stress distribution between PDMS and 3C-SiC, as shown in **Figure S14**.

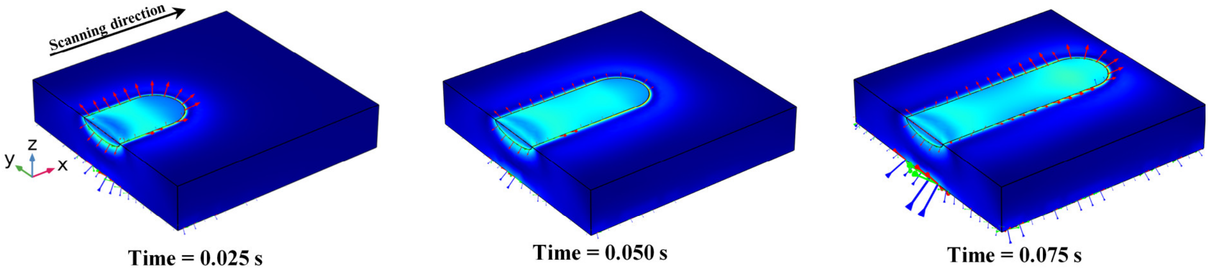

Figure S14. The principal stress line distributions of PDMS and SiC during continuous laser pyrolysis (Time = 0.025, 0.050, and 0.075 s).

3. Supplementary References

1. Anselmo, L.; Pardini, C., Long-Term Evolution of High Earth Orbits: Effects of Direct Solar Radiation Pressure and Comparison of Trajectory Propagators. *ISTI/CNR Technical Report* **2007,** *29*.
2. Moufekkir, F.; Moussaoui, M.; Mezrhab, A.; Naji, H.; Lemonnier, D., Numerical Prediction of Heat Transfer by Natural Convection and Radiation in an Enclosure Filled with an Isotropic Scattering Medium. *Journal of Quantitative Spectroscopy and Radiative Transfer* **2012,** *113*, 1689-1704.
3. Burns, G. T.; Taylor, R. B.; Xu, Y.; Zangvil, A.; Zank, G. A., High-Temperature Chemistry of the Conversion of Siloxanes to Silicon Carbide. *Chemistry of materials* **1992,** *4*, 1313-1323.
4. Camino, G.; Lomakin, S.; Lazzari, M., Polydimethylsiloxane Thermal Degradation Part 1. Kinetic Aspects. *Polymer* **2001,** *42*, 2395-2402.
5. Camino, G.; Lomakin, S.; Lageard, M., Thermal Polydimethylsiloxane Degradation. Part 2. The Degradation Mechanisms. *Polymer* **2002,** *43*, 2011-2015.
6. Shin, J.; Ko, J.; Jeong, S.; Won, P.; Lee, Y.; Kim, J.; Hong, S.; Jeon, N. L.; Ko, S. H., Monolithic Digital Patterning of Polydimethylsiloxane with Successive Laser Pyrolysis. *Nature Materials* **2021,** *20*, 100-107.
7. Larruquert, J. I.; Pérez-Marín, A. P.; García-Cortés, S.; Rodríguez-de Marcos, L.; Aznárez, J. A.; Méndez, J. A., Self-Consistent Optical Constants of Sic Thin Films. *JOSA A* **2011,** *28*, 2340-2345.
8. Zhang, X.; Qiu, J.; Li, X.; Zhao, J.; Liu, L., Complex Refractive Indices Measurements of Polymers in Visible and near-Infrared Bands. *Applied optics* **2020,** *59*, 2337-2344.
9. Poulain, G.; Blanc, D.; Kaminski, A.; Semmache, B.; Lemiti, M. In *Modeling of Laser Processing for Advanced Silicon Solar Cells*, Excerpt from the Proceedings of the COMSOL Conference, 2010.
10. Saleh, B. E.; Teich, M. C., Fundamentals of Photonics John Wiley & Sons. *Inc., Hoboken NJ* **1991,** *2*.
11. Marshall, W. J., Two Methods for Measuring Laser Beam Diameter. *Journal of Laser Applications* **2010,** *22*, 132-136.
12. Darif, M.; Semmar, N.; Orléans Cedex, F. In *Numerical Simulation of Si Nanosecond Laser Annealing by Comsol Multiphysics*, Proceedings of the COMSOL Conference 2008 Hannover, 2008; pp 567-571.
13. Wang, W.; Lu, L.; Xie, Y.; Wu, W.; Liang, R.; Li, Z.; Tang, Y., Controlling the Laser Induction and Cutting Process on Polyimide Films for Kirigami-Inspired Supercapacitor Applications. *Science China Technological Sciences* **2021,** *64*, 651-661.
14. Ruan, X.; Wang, R.; Luo, J.; Yao, Y.; Liu, T., Experimental and Modeling Study of Co2 Laser Writing Induced Polyimide Carbonization Process. *Materials & Design* **2018,** *160*, 1168-1177.
15. Zhang, Q.; Ma, Y.; Cui, C.; Chai, X.; Han, S.., Experimental Investigation and Numerical Simulation on Welding Residual Stress of Innovative Double-Side Welded Rib-to-Deck Joints of Orthotropic Steel Decks. *Journal of Constructional Steel Research* **2021,** *179*, 106544.
